# Influence of chain length and branching on poly(ADP-ribose)–protein interactions

**DOI:** 10.1093/nar/gkac1235

**Published:** 2023-01-10

**Authors:** Tobias Löffler, Annika Krüger, Peyman Zirak, Martin J Winterhalder, Anna-Lena Müller, Arthur Fischbach, Aswin Mangerich, Andreas Zumbusch

**Affiliations:** Department of Chemistry, Universität Konstanz, Konstanz D-78457, Germany; Department of Biology, Universität Konstanz, Konstanz D-78457, Germany; Department of Chemistry, Universität Konstanz, Konstanz D-78457, Germany; Department of Chemistry, Universität Konstanz, Konstanz D-78457, Germany; Department of Chemistry, Universität Konstanz, Konstanz D-78457, Germany; Department of Biology, Universität Konstanz, Konstanz D-78457, Germany; Department of Biology, Universität Konstanz, Konstanz D-78457, Germany; Institute of Nutritional Science, University of Potsdam, D-14558 Nuthetal, Germany; Department of Chemistry, Universität Konstanz, Konstanz D-78457, Germany

## Abstract

Hundreds of proteins interact with poly(ADP-ribose) (PAR) via multiple PAR interaction motifs, thereby regulating their physico-chemical properties, sub-cellular localizations, enzymatic activities, or protein stability. Here, we present a targeted approach based on fluorescence correlation spectroscopy (FCS) to characterize potential structure-specific interactions of PAR molecules of defined chain length and branching with three prime PAR-binding proteins, the tumor suppressor protein p53, histone H1, and the histone chaperone APLF. Our study reveals complex and structure-specific PAR–protein interactions. Quantitative *K*_d_ values were determined and binding affinities for all three proteins were shown to be in the nanomolar range. We report PAR chain length dependent binding of p53 and H1, yet chain length independent binding of APLF. For all three PAR binders, we found a preference for linear over hyperbranched PAR. Importantly, protein- and PAR-structure-specific binding modes were revealed. Thus, while the H1-PAR interaction occurred largely on a bi-molecular 1:1 basis, p53—and potentially also APLF—can form complex multivalent PAR–protein structures. In conclusion, our study gives detailed and quantitative insight into PAR–protein interactions in a solution-based setting at near physiological buffer conditions. The results support the notion of protein and PAR-structure-specific binding modes that have evolved to fit the purpose of the respective biochemical functions and biological contexts.

## INTRODUCTION

Poly(ADP-ribose) (PAR) is a nucleic acid-like biopolymer, which is synthesized by certain members of the family of ‘ADP-ribosyl transferases diphtheria toxin-like’ (ARTDs, aka PARPs), such as PARP1, PARP2 and the tankyrases PARP5a/b. These enzymes act as ‘writers’ of poly(ADP-ribosyl)ation (PARylation) (1). Upon catalytic activation, PARPs use NAD^+^ as a substrate to synthesize PAR, which can be attached to specific amino acids of target proteins, including serines, glutamates, aspartates and tyrosines, with the release of nicotinamide as a by-product (2–6). In general, PARylation fulfils important roles in various cellular processes, such as DNA damage response, transcription, energy metabolism, as well as regulation of cell death (2–5). Of note, several highly potent small-molecule inhibitors of PARPs have been approved for clinical use in oncology following the concept of synthetic lethality, and a plethora of clinical trials have been performed to test their use in combination therapy with other DNA damaging chemotherapeutics as well as with immune checkpoint inhibitors (7–9). PAR molecules can be of heterogeneous chain lengths consisting of up to 200 ADP-ribose units, which are linked via 1″→2′ ribose-ribose bonds. Furthermore, PAR can be branched at about every 20–50 subunits via 1′→2′ ribose-ribose linkages (4,5,10).

PARylation is a highly dynamic and completely reversible post-translational modification, since several ‘erasers’ of PARylation exist, which can remove PAR from proteins. Thus, poly(ADP-ribose) glycohydrolase (PARG), certain Nudix hydrosylases (NUDTs) and ADP-ribose hydrolase 3 (ARH 3) can degrade PAR molecules (11). Furthermore, the terminal ADP-ribose protein glycohydrolase 1 (TARG1) can cleave off whole PAR molecules from proteins, and TARG1, ARH3, and MacroD1/2 can remove the most proximal protein-bound ADP-ribose moieties (11). Apart from enzymes that covalently attach or remove PAR from proteins, also various ‘readers’ of PARylation exist. These interact with PAR molecules with high affinity via a spectrum of different PAR binding modules, such as a loosely conserved PAR-binding motif (PBM), consisting of a cluster of ∼20 basic and hydrophobic amino acids, as well as the WWE domain, macrodomains, and the PAR binding zinc finger motif (PBZ) (10,12–14). Covalent PARylation as well as non-covalent binding of PAR is thought to regulate biophysical properties of proteins and thereby to control their functions in cells in a highly dynamic and spatio-temporally defined manner (15,16). There is evidence supporting the existence of a so-called PAR-code, following the hypothesis that PAR chains of specific length and branching frequency exhibit specific functions (10,14,16,17). In favor of this notion, some proteins such as, e.g. histones, p53 or XPA, show a preference for binding PAR of specific chain length (10,18–23). Moreover, PAR branching may serve as a recognition site for non-covalent protein binding (19,24,25). PAR branching may also lead to stabilization of the PAR structure, since branching points are not the preferential targets for PARG-dependent PAR degradation and may therefore serve as a means for the regulation of polymer half-life (17,26,27). Interestingly, it has been reported that PARP2 can be activated by non-covalent binding to PAR, which subsequently catalyzes the synthesis of PAR chains with higher branching frequencies (25). On the contrary, TNKS1 appears to produce mainly linear PAR chains (28). Despite such initial insight into PAR-structure-specific biochemistry, our understanding of the relevance of PAR chain length and degree of branching, in particular with regards to non-covalent protein interactions, is just emerging (10).

**Figure 1.**
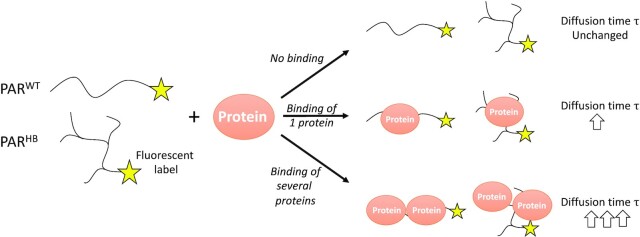
Simplified scheme visualising potential PAR–protein binding scenarios and resulting consequences on the diffusion time τ measured via FCS. For details, see text. PAR^WT^ indicates PAR synthesized by wildtype PARP1 whereas PAR^HB^ relates to PAR synthesized by the hyperbranching variant PARP1^Y986H^.

In this study, we elucidate the role of PAR chain length and branching in non-covalent protein binding. We enzymatically synthesized, purified, and characterized distinct PAR molecules, which were structurally defined with respect to their chain length and branching, and investigated their binding characteristics with model proteins. As an analytical method, we used fluorescence correlation spectroscopy (FCS). FCS is a spectroscopic technique, which is well established for determining diffusion coefficients of fluorescent molecular species (29). Since translational diffusion coefficients are inversely proportional to the radius of the diffusing species, FCS based measurements of diffusion coefficients give insight into the interaction of proteins with binding partners including binding stoichiometries and dissociation constants *K*_d_ (30) (Figure 1). FCS is highly suitable to investigate the interaction between PAR and the respective proteins, since measurements can be performed in aqueous solution and because it allows the observation of interactions with *K*_d_ values in the nanomolar range.

As model proteins, we employed the strong PAR binders p53, histone H1, and the histone chaperone APLF (aprataxin polynucleotide kinase (PNK)-like factor). The tumour suppressor p53 acts as a transcription factor, which can swiftly be activated in response to a series of different cellular stressors in order to control cell cycle regulation and cell death (31). Recently, we have shown that p53 binds PAR via its intrinsically disordered C-terminal domain (CTD) harbouring a sequence that resembles the classical PBM. PAR interacts with the CTD with high affinity, which induces distinct changes in the structure of p53, in turn targeting p53 for covalent PARylation by PARP1. On a functional level, the p53-PAR interaction has several substantial implications, such as regulating p53′s DNA binding properties, its protein-binding interactome, as well as gene transcription and choice of DNA repair pathways (21,22,32).

The linker histone H1 binds to the nucleosome, which contains the core histones H2-H4, to form chromatosomes, which help to regulate higher-order chromatin structures (33). Histone H1 is both a target for covalent PARylation (34,35) as well as a non-covalent PAR binder (19,36). Of all histones, histone H1 represents the strongest non-covalent PAR binder (19,37,38) with a chain length dependent preference for longer PAR polymer (36). The exact binding mode is unknown, but it was shown that the bulk of the PAR binding is mediated by its basic C-terminal domain (19), presumably via a PBM-like motif (39). Functionally, it was shown that PARP1 can displace H1 from chromatin and that reciprocal binding of PARP1 and histone H1 at gene promoters participate in the regulation of transcription (40,41).

APLF acts as a histone chaperone with endo and exonuclease activities, harbouring an FHA domain and unique zinc fingers, which were shown to participate in DNA double strand break repair, in particular non-homologous end-joining (NHEJ) (42–48). Collectively, biochemical and structural evidence indicates that the tandem PBZs located in APLF bind PAR cooperatively with very high affinity (49–51). It has been reported that the tandem PBZ motif of APLF shows a preference for PAR branching points, thereby regulating chromatin remodelling and removal of histone H3 in response to DNA damage (25).

## MATERIALS AND METHODS

### Purification of PARP1 variants and PAR synthesis

Purification of PARP1^WT^ (producing wildtype PAR, i.e. PAR^WT^) and PARP1^Y986H^ (producing hyperbranched PAR, i.e. PAR^HB^) and subsequent synthesis of PAR was performed as described previously (21,52). Bacterial pellets [*Escherichia coli* strain Rosetta 2 (DE3)] from 2 l cultures were resuspended in lysis buffer (25 mM HEPES pH 8.0, 500 mM NaCl, 0.5 mM DTT, 10 mM benzamide) supplemented with 0.1 % NP-40, complete EDTA-free protease inhibitor cocktail (Roche), and 1 mg/ml lysozyme (Sigma-Aldrich), and sonicated four times for 20 s. Then, 5 μg/ml DNase I (Roche) was added and incubated for 1 h. Cell debris was removed by centrifugation (68 000 × g, 2 h), the supernatant was filtered (syringe filter, 0.2 μm, Corning) and loaded onto a HisTrap HP column (GE Healthcare). After washing with 10 ml of 1 M NaCl, PARP1 was eluted with 30 ml of 500 mM imidazole. The elution fraction was diluted to a final NaCl concentration of 375 mM NaCl with no-salt heparin buffer (50 mM Na-phosphate pH 7.0, 1 mM EDTA) and loaded onto a heparin HP column (GE Healthcare). PARP1 was eluted by gradually increasing the NaCl concentration up to 1 M (30 ml). PARP1 containing fractions were concentrated and buffer was exchanged (50 mM Tris pH 8, 150 mM NaCl, 0.5 mM DTT) via centrifugal filters (Amicon Ultra 15, 10 kDa MWCO).

For PAR synthesis, the total amount of recombinant PARP1 protein from one purification was incubated at 37°C in a buffer containing 100 mM Tris (pH 7.8), 10 mM MgCl_2_, 1 mM DTT supplemented with 300 μg/ml histone H2a, 50 μg/ml *EcoR*I linker oligonucleotide (GGAATTCC), and 1 mM NAD^+^ in a total volume of 10 ml. After 45 min, the reaction was stopped with 10 ml 20% ice-cold TCA. After 15 min of incubation on ice, the sample was centrifuged (10 min, 9000 × g, 4°C). The pellet was washed twice with 1 ml ethanol and finally dried using a nitrogen flow. To separate PAR from proteins the pellet was resuspended and incubated for 10 min at 37°C at alkaline conditions (0.5 M KOH, 50 mM EDTA). Then, the pH was immediately adjusted to 7.5–8. The sample was supplemented with 50 mM MgCl_2_ and 50 μg/ml DNase I and incubated for 2 h at 37°C. Proteins were digested overnight by the addition of 1 mM CaCl_2_ and 50 μg/ml proteinase K. The next day, PAR was purified via two cycles of phenol/chloroform/isoamylalcohol (25:24:1) (Roth) extraction. In a last step, residual phenol was removed via mixing the sample with chloroform. The aqueous phase was collected, and PAR was precipitated by the addition of 70% ethanol and incubation at –20°C overnight. The pellet was dissolved in water and the PAR concentration was determined by measuring the absorbance at 258 nm (ϵ = 13.500 M^−1^cm^−1^). Concentrations determined are 2.0 mM for PAR^WT^ and 4.8 mM for PAR^HB^.

### Size-fractionation of PAR

PAR chains were fractionated by chain lengths using an HPLC (Agilent 1100) equipped with a DNA Pac PA-100 column (Thermo Fisher) as described previously (21). The column was equilibrated with 25 mM Tris (pH 9) and 100 μl PAR was loaded on the column, respectively. Elution was performed by increasing the NaCl concentration (25 mM Tris, pH 9, 1 M NaCl) using the following gradient settings: 0–3 min 20%, 3–20 min 35%, 20–40 min 42%, 40–70 min 47%, 70–110 min 53%, 110–120 min 61%, 120–131 min 70%, 131–132 min 100%. Fractions were concentrated to a final volume of 20–60 μl and buffer was exchanged (20 mM Na-phosphate buffer, pH 7.0) via centrifugal filters (Vivaspin 2, 2 kDa MWCO).

### Preparation of Ado, R-Ado and R_2_-Ado standards

To obtain a high yield of R_2_-Ado, PAR^HB^ was produced by the PARP1-Y986H variant as described above. Purified PAR was digested to Ado, R-Ado and R_2_-Ado by incubation with phosphodiesterase (PDE) and alkaline phosphatase (AP) for 3 h at 37°C (i.e. 500 μM PAR, 1 mM MgAc_2_, 25 mM NH_4_Ac, 0.045 U/μl AP and 0.002 U/μl PDE). Afterwards, the incubation mix was loaded on a NANOSEP 10K Omega column and centrifuged at room temperature for 5 min and 15,700 x *g* for the removal of PDE and AP. The flow through was aliquoted into 20 μL aliquots and stored at –20°C until further use.

Ado, R-Ado and R_2_-Ado were separated via HPLC. Therefore, the PAR digest was loaded on a Hydro RP 80A, 250 × 4.6 mm column and chromatography was conducted at 40°C, with a flow rate of 0.7 ml/min and the gradient as shown in Supplementary Table S1:

Fractions containing Ado, R-Ado and R_2_-Ado were collected at their empirically determined retention times (Supplementary Figure S1), desiccated under vacuum, and redissolved in 50 μl H_2_O in case of Ado and R-Ado, or 70 μl H_2_O in case of R_2_-Ado. Due to remaining Ado contamination, the R_2_-Ado fraction was subjected to a second round of purification as described above. Concentrations were determined via absorption measurement at 259 nm. Aliquots of 1 μM Ado, 10 μM R-Ado and 1 μM R_2_-Ado were prepared respectively, shock-frozen in liquid nitrogen and stored at –80°C. Mass spectrometric measurements of standard curves of Ado, R-Ado and R_2_-Ado were performed essentially as described before (53,54).

### Mass spectrometric characterization of PAR

HPLC-fractionated PAR chains were digested with alkaline phosphatase and phosphodiesterase to nucleosides as described above and then subjected to UPLC–MS/MS analysis, essentially as described previously (53,54). Using external standard curves, the absolute amounts of Ado, R-Ado and R_2_-Ado were determined, and the branching ratio (BR) and average polymer length (APL) were calculated by:


\begin{eqnarray*} BR\ \left[ \% \right]\ = \ \frac{{{R}_2Ado}}{{{R}_2Ado + RAdo + Ado}}{\rm{\ }} \times 100 \end{eqnarray*}

\begin{eqnarray*} APL\ = \ \frac{{Ado + RAdo + {R}_2Ado}}{{Ado - {R}_2Ado}} \end{eqnarray*}

### Fluorescence labelling of PAR for FCS

PAR was labelled via Alexa Fluor 647 hydroxylamine (Thermo Fisher), which forms an oxime bond at the terminal ribose of PAR. Labelling was performed after size-fractionation via HPLC, because the fluorescent label significantly affected retention times. Thus, 25 μl of PAR^WT^ or 20 μl of PAR^HB^ were incubated with 0.5 mM Alexa Fluor 647 hydroxylamine (25 mM stock solution in DMSO) and 0.5 μl aniline in a total volume of 50 μl of 100 mM Na-phosphate (pH 7.0) overnight at room temperature. Addition of aniline for catalysis significantly improved labelling rates (55,56). Excess labelling dye was removed via Micro Bio-Spin 6 columns (Bio-Rad) using 20 mM Na-phosphate buffer (pH 7.0). Purification was repeated three times and final PAR concentrations were determined by measuring the absorbance at 258 nm. Labelled PAR fractions were analysed on a 20% polyacrylamide sequencing gel by loading of 50 pmol of each PAR fraction and 100 pmol of unfractionated PAR (amounts given in ADP-ribose subunits), respectively. After fluorescence read-out, silver staining was performed. Chain lengths were determined via Orange G (corresponding to an ADP-ribose monomer), bromophenol blue (corresponding to an ADP-ribose 8-mer), and xylene cyanol (corresponding to an ADP-ribose 20-mer).

To determine the labelling efficiencies, we analysed individual PAR samples at concentrations of 10 nM by FCS. The concentration of labelled PAR chains was derived from the amplitude of the FCS curves, which is inversely proportional to the concentration of the dye. Labelling efficiencies were then calculated by relating the concentrations of labelled PAR chains thus obtained to the 10 nM of PAR chains used in the FCS experiment. We obtained labelling efficiencies of 18–36% for PAR^WT^ and of 2–4% for PAR^HB^.

### Recombinant PAR-binding proteins

Purification of human p53^WT^, p53^PBM4^ (R363A, K370A, R379A, K381A) (22) and p53^MONO^ (L344P) (57) were performed as described previously (21). Bacterial pellets of *E. coli* strain BL21(DE3) from 2 l cultures were resuspended in lysis buffer (50 mM Na-phosphate pH 8, 300 mM NaCl, 10 mM β-mercaptoethanol) supplemented with complete EDTA-free protease inhibitor cocktail (Roche) and 1 mg/ml lysozyme (Sigma-Aldrich), and sonicated four times for 20 s. Then, 5 μg/ml DNase I (Roche) was added, and samples were incubated for 1 h. Cell debris was removed by centrifugation (20 000 × g, 30 min) and the supernatant was filtered (syringe filter, 0.45 μm, Corning). After adding 10 mM imidazole, the filtrate was loaded onto a HisTrap HP column (GE Healthcare). Following washing with 10 ml of 20 mM imidazole, p53 was eluted with 30 ml of 500 mM imidazole. The polyhistidine-tag was removed via thrombin digestion (1 U/ml) during dialysis overnight (20 mM Na-phosphate pH 8, 100 mM NaCl, 1 mM β-mercaptoethanol). Thrombin digestion was stopped by the addition of 0.1 mg/ml Pefablock (Roche). Next, the samples were loaded onto a heparin HP column (GE Healthcare). Weakly bound proteins were removed by washing with 5 ml 200 mM NaCl. Elution was performed by an NaCl gradient up to 1 M (30 ml). p53-containing fractions were concentrated to 2–3 ml via centrifugal filters (Amicon Ultra 15, 10 kDa MWCO) and applied to size exclusion chromatography using a HiLoad 16/600 Superdex 200 column (GE Healthcare) using a buffer of 50 mM Tris pH 7.4, 300 mM NaCl, 1 mM DTT. The flow rate was set to 0.3 ml/min. p53 eluted in two peaks, of which only the fraction of the second peak was concentrated using a centrifugation device (Amicon Ultra 4, 10 kDa MWCO). The fraction of the first peak was discarded, as it contains high molecular-weight aggregates.

Recombinant human histone H1 and APLF were purchased from BPS Bioscience and Origene Technologies, respectively.

### Sample preparation

p53 variants, histone H1, and APLF were diluted in Tris buffer (50 mM Tris, pH 7.4, 150 mM NaCl) with 3 mM DTT and 0.05% Tween20 and incubated for 1 h at room temperature prior to FCS experiments. All experiments were performed in μ-slides (ibidi) using 20 μl of the sample solution. The experimental setup was first tested by studying the well-known interaction of p53 with DNA response element p21 (DNA_RE_; Cy5-labeled double stranded p53 response element from the p21 promotor, i.e. RE_p21_, (FWD: Cy5-CGAGGAACATGTCCCAACATGTTGCTCGAG) and a scrambled version of it, i.e. RE_p21_^scr^ (FWD: Cy5-GTCGCTGACCCGAGACTAGGCGTTCAAATA). The concentrations of PAR or RE_p21_ were always kept constant at 10 nM with labelling degrees between 2% and 32% for PAR and close to 100% for RE_p21_. The dilution series was started with a 5 μM solution of the respective protein. The solution was diluted 12 times 1:1 down to the lowest concentration of 1.22 nM. As a control, the buffer containing PAR only was measured in all experiments. For the logarithmic presentation of the data, results of the latter measurements were plotted at abscissa values of 0.1 nM. Since bigger relative mass changes are easier to analyse, we always labeled the smaller interaction partner. All experiments were carried out in two independent experiments, each in technical triplicates (*n* = 6).

### Optical setup

FCS and photon counting histogram (PCH) experiments were performed using a custom-built confocal epifluorescence microscopy setup. A fibre-coupled 635-nm diode laser (LDH-D-C-635, PicoQuant) served as the excitation source. After collimation with an air objective (10×, NA 0.25, Leica) and spectral filtering (BrightLine HC 630/20, Semrock), the beam was expanded to overfill the back aperture of a water/glycerol microscope objective (63×, NA 1.3, Zeiss) mounted on a wide-field microscope (Axiovert 200, Zeiss). Fluorescence emission collected with the same objective was separated from the excitation light using a dichroic mirror (640dcxr, Chroma). The fluorescence signal was focused onto a 50 μm diameter pinhole and filtered twice to remove background from the excitation laser (NF01-633U-25 and LP 647 RU, Semrock). After filtering, the signal was split by a 50:50 beam splitter cube and focused onto two avalanche photodiodes (APD; SPCM AQR 14, Perkin Elmer). Breakdown signals of the APDs were suppressed with a short pass filter (ET 7002P8, Chroma) in one detection arm and a bandpass filter (BP 684/24, Semrock) in the other. Fast electronics (HydraHarp, PicoQuant) were used to record the signals. For all measurements, the laser was operated in continuous wave mode. Excitation powers used at the back aperture of the microscope objective were set to 120 μW for FCS and 47 μW for PCH.

### FCS fitting

Time traces for FCS analysis were recorded over 60 s. For the calculation and the fitting of the second order autocorrelation function


\begin{eqnarray*} {g}^{\left( 2 \right)}\ \left( \tau \right) = \ \frac{{\langle I\left( t \right)I\left( {t + \tau } \right)\rangle }}{{{{\langle I\left( t \right)\rangle }}^2}} \end{eqnarray*}

with the fluorescence intensities *I*(*t*) and *I*(*t*+τ) as the fluorescence intensity at time *t* and after a time shift τ, respectively, a commercial software was used (SymphoTime, PicoQuant). To derive diffusion times from the autocorrelation data, we employed


\begin{eqnarray*} {g}^{\left( 2 \right)}\ \left( \tau \right) = \ \frac{{{g}_0}}{{\left[ {1 + \frac{\tau }{{{\tau }_d}}} \right]{{\left[ {1 + \frac{\tau }{{{\tau }_d{\kappa }^2}}} \right]}}^{1/2}}} \end{eqnarray*}

with the amplitude *g*_0_, the diffusion time ${\tau }_d$, and the length to diameter ratio of the focal volume $\kappa$. For the fitting, data obtained for time shifts of 0.1 ms ≤ ${\tau }_d$ ≤ 1000 ms were used. The limit at short times was chosen in order to exclude any influence of orientation dynamics between the fluorophores or photochemical triplet population dynamics of the dyes on the observed fluorescence fluctuations. As a calibration measurement, diffusion times for Atto655-carboxylic acid (AttoTec) for which a diffusion coefficient of *D* = 4.04 × 10^−6^ cm^2^/s in aqueous buffer is known (58), were determined. Additionally, the diffusion coefficients for the different labelled PAR chains used in our experiments were measured once. These values later served as reference points for the determination of the confocal volumes at every measurement day. Binding curves were obtained by plotting diffusion times as a function of experimentally used protein concentrations. Due to their radius dependence, diffusion times could be used to estimate molecular masses of the diffusing species. To this end, we calibrated FCS measurements with diffusion measurements of globular proteins that are commonly used in size exclusion chromatography as size standards (Supplementary Figure S2). With the calibration curve, an upper limit for the mass of the diffusing species could be determined, since all deviations from the spherical shape lead to longer diffusion times and therefore to higher calculated masses. (59)

Concentration dependent diffusion time measurements based on FCS data were used to determine *K*_d_ values. The procedure employed is described in detail in the supporting information (Supplementary Figures S3–S5).

### Photon counting histogram (PCH) fitting and statistics

We were interested in addressing the issue of whether especially longer PAR chains could serve as building blocks of larger aggregates in which p53 binds to several different PAR chains. In this case, one would observe diffusing species with more than one fluorescent label. The occurrence of such multiply labelled species was investigated using PCH (60). Time traces for PCH analysis were recorded for 300 s and analysed using commercial software (FFS Data Processor). The probability for observing diffusing species with several fluorescent labels decreases rapidly with the average number of fluorescent labels per PAR chain. Since our PAR labelling degrees for the different chain types and lengths varied between 2 and 36%, we used a model considering only either singly or multiply labelled species. A first order correction term for the shape of the focus was employed (61). Furthermore, the possible detection of after-pulses from the APDs was incorporated into the data analysis. For the estimation of the composition of the PAR/p53 complex, we assumed that the fitting results yield a number and brightness for the complexes with only one dye (*N*_1_; *q*_1_) and one number and brightness for all multiply labelled species (*N*_*m*_; *q*_*m*_). Under these conditions and for a given labelling degree *L* of the PAR chains, we calculated the probability of having one labelled species *N*_1_ as a function of the total number *P* of PAR chains in the complex:


\begin{eqnarray*} {N}_1\left( P \right)\ = \ L \cdot \left( {{{\left( {1 - L} \right)}}^{\left( {P - 1} \right)}} \right) \cdot P \end{eqnarray*}

In general, the probability *N_x_*(*P*) of finding a complex consisting of *P* PAR chains and containing *x* labelled PAR chains is calculated to be


\begin{eqnarray*} {N}_x\ \left( P \right) = {L}^x\ \cdot ({\left( {1 - L} \right)}^{\left( {P - x} \right)} \cdot \left( {\frac{{P!}}{{x! \cdot \left( {P - x} \right)!}}} \right) \end{eqnarray*}

The probability *N_m_*(*P*) for complexes that contain more than one dye then is given by:


\begin{eqnarray*} {N}_m\left( P \right)\ = \ 1 - {\left( {1 - L} \right)}^P - {N}_1 \end{eqnarray*}

Assuming that every dye molecule in the complex added the same overall emission intensity, one can derive the average brightness *q_m_*(*P*) of the complex also when it contains multiple dyes:


\begin{eqnarray*} {q}_m\ \left( P \right) = \ \mathop \sum \limits_{x\ = \ 2}^P x \cdot {N}_x\left( P \right) \end{eqnarray*}

With these equations, we calculated the total number of PAR chains P from the ratios of *N*_1_ to *N_m_* and *q*_1_ to *q_m_* from our experiments.

## RESULTS AND DISCUSSION

### Diffusion properties of PAR chains with different lengths and branching

**Figure 2.**
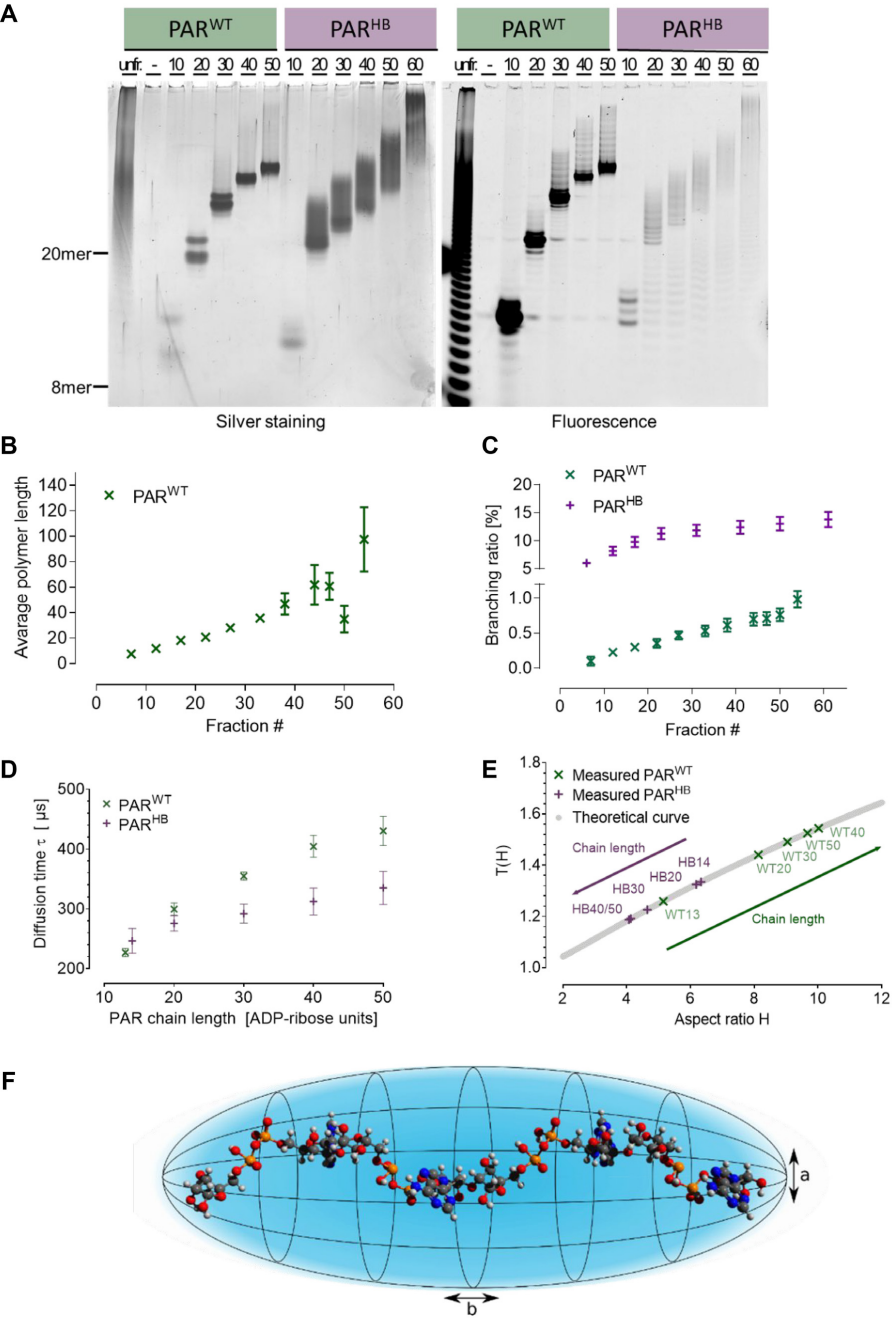
Characterization of PAR chains with different lengths and branching by FCS. (**A**) Native PAGE of Alexa Fluor 647-labelled PAR^WT^ and PAR^HB^ fractions (left: silver staining; right: fluorescence readout). Unfractionated (unfr.) PAR was loaded as control. (**B**) Determination of average chain length of fractionated PAR^WT^ measured by mass spectrometry as described in the methods section. (**C**) Mass spectrometric determination of PAR branching ratios of fractionated PAR^WT^ and PAR^HB^ as described in the methods section. For HPLC chromatogram, sequencing gel as well as Ado, R-Ado, and R_2_-Ado UPLC-MS/MS standard curves please refer to Supplementary Figure S1. Experiments shown in (B) and (C) were performed in three independent experiments, mean ± SEM. (**D**) Diffusion times $\tau$ for PAR^WT^ (green crosses) and PAR^HB^ (purple crosses) as a function of the number of ADP-ribose subunits. All measurements were performed at a concentration of 10 nM of PAR. (**E**) Aspect ratios of the different PAR chains as indicated derived under the assumption of a prolate, ellipsoidal shape. T(H) describes the deviation of diffusion times between spherical and prolate ellipsoidal objects, where H = b/a is the aspect ratio of the ellipsoid. HB indicates hyperbranched, WT, wildtype. (**F**) Prolate ellipsoidal shape model of a diffusing PAR chain. The negative charges of the phosphate residues prevent a globular shape of the dissolved PAR chains. For analyses in this study, a prolate ellipsoidal shape with a long semiaxis ‘b’ and a short semiaxis ‘a’ is used as a model for the hydrodynamic shape of PAR chains diffusing in aqueous solution.

Based on our previous study by Aberle et al., which revealed PAR-structure-specific cellular stress response phenotypes (17), here, we elucidated the impact of PAR structure on the non-covalent interaction characteristics with selected PAR binding model proteins in controlled in-vitro settings. To this end, we generated PAR with distinct branching frequencies using recombinant PARP1 variants and fractionated PAR samples according to chain lengths (Supplementary Figure S1). Wildtype PARP1 was used to obtain ‘wildtype’ PAR (PAR^WT^), which was demonstrated to exhibit ∼1% branching points (62), and the variant PARP1^Y986H^ was used to obtain hyperbranched PAR (PAR^HB^), which was demonstrated by us and others to exhibit ∼15-fold higher branching ratios (63,64). In order to form a solid basis for subsequent FCS experiments we further characterized the size-fractionated PAR by high-resolution polyacrylamide electrophoresis and mass spectrometry. First, size-fractionated PAR chains were fluorescently labelled via Alexa Fluor 647 hydroxylamine and analysed via sequencing gels (Figure 2A). We obtained fractions of labelled PAR molecules consisting of approximately 10, 20, 30, 40 and 50 ADP-ribose units displaying a distinct size distribution. It is important to note that the indicated sizes are approximate numbers based on the size markers Orange G (corresponding to an ADP-ribose monomer), bromophenol blue (corresponding to an ADP-ribose 8-mer), and xylene cyanol (corresponding to an ADP-ribose 20-mer) as published previously (65). Assuming a maximum chain length of ∼50 ADP-ribose units and a branching frequency of ∼1%, it was estimated that PAR^WT^ predominantly consisted of linear PAR molecules, whereas the corresponding PAR^HB^ fraction exhibited ∼7–8 branching points per molecule. This difference in branching ratios was also represented in the sequencing gel analysis, since PAR^WT^ displayed sharp bands of individual molecules, whereas PAR^HB^ bands appeared ‘blurry’, which presumably reflected the higher structural complexity of PAR^HB^ compared to PAR^WT^ (Figure 2A). A comparison between silver staining and the fluorescence readout revealed that fluorescence labelling affects migration of PAR chains, resulting in a slight upwards shift of the respective labelled molecules. This also shows that only a fraction of PAR molecules was fluorescently labelled as discussed in Materials and Methods.

To further characterize the size-fractionated PAR molecules we performed an independent set of experiments with an advanced version of our previously published mass spectrometric platform using external standard curves of purified Ado, R-Ado, and R_2_-Ado (Figure 2B, C and Supplementary Figure S1). [For details of the calculation of average polymer length and branching ratios, please refer to the methods section.] These data show a steady increase of chain length with each fraction of PAR (Figure 2B) and confirmed the results on chain length estimations obtained by HPLC chromatograms and PAGE analysis (Figure 2A, B and Supplementary Figure S1). For technical reasons, we were not able to determine the absolute chain length of individual fraction of PAR^HB^ by mass spectrometry. The reasons for this are not entirely clear, however presumably depend on the low abundance and signal intensity of R_2_-Ado in comparison to Ado and R-Ado. This is not a problem for the chain length determination of PAR^WT^, since here R_2_-Ado amounts are negligible and therefore do not contribute substantially in the calculations. Since HPLC-based PAR fractionation is performed via an anion-exchange column and therefore retention times largely depend on molecule charge, in our view a direct comparison of chain length of PAR^WT^ and PAR^HB^ of the corresponding fractions appears to be a valid assumption. We further characterized the PAR fractions by mass spectrometry with regards to their branching ratios (Figure 2C). These results revealed a chain-length dependent increase of branching in PAR^WT^ from 0.1% to 1%. In contrast, branching ratios of PAR^HB^ ranged from 6% for early fractions up to ∼14% for late fractions.

With PAR chains of different length and branching ratios at hand, we first investigated their diffusion properties using FCS. In general, FCS records emission intensity fluctuations in the focus of a confocal fluorescence microscope. In dilute solutions of fluorescent molecules with concentrations in the nanomolar range, fluctuations of the intensity I(t) are caused by the diffusion of fluorescent molecules in and out of the microscope focus or by photophysical processes leading to fluorescence intermittencies (66). Only diffusion related fluctuations of fluorescence intensities were of relevance here. FCS data analysis was based on the calculation of the autocorrelation function g^(2^^)^(τ) from the measured intensity signal (an exemplary data set of second order autocorrelation functions is shown in Figure 4A and Supplementary Figures S3 and S4). The fitting of g^(2)^(*τ*) yields the diffusion time ${\tau }_D$, which for our experimental setting is related to the diffusion coefficient *D* by $D\ = \ \omega _{xy}^2/4{\tau }_D$, where *ω_xy_* is the lateral radius of the detection volume. For small molecules, usually a spherical shape of the diffusing molecule is assumed. In the regime of free, Brownian motion, the diffusion coefficient *D* is given by $D\ = {k}_B\ T/{f}_{sphere}$ with the Boltzmann constant *k_B_*, temperature *T*, and Stokes’ friction coefficient of a sphere ${f}_{sphere} = \ 6\pi \eta r$ with the viscosity *η* and the hydrodynamic radius r of the diffusing fluorescent species. The size of the diffusing molecule therefore directly relates to the experimentally determined diffusion times ${\tau }_D$.

To determine the diffusion times of fluorescently labelled PAR chains in dependence on their length and branching, we performed FCS experiments of PAR at concentrations of 10 nM. For both linear (i.e. PAR^WT^) and hyperbranched PAR (i.e. PAR^HB^), we found that the diffusion times increased with chain length (Figure 2D). As pointed out above, in most analyses diffusing molecules were assumed to be spherical in shape. Under this assumption, one expects to observe the same dependence of the diffusion times on PAR chain length for both types of PAR chains. Yet, with increasing chain length the diffusion times for PAR^WT^ increased more than those of PAR^HB^. This discrepancy can be explained by the increasing non-spherical shape of PAR^WT^ with increasing chain length. Since ADP-ribose subunits carry negative charges on the phosphate residues, electrostatic repulsion probably prevents the formation of a densely folded 3D structure. Long PAR chains therefore presumably do not adopt a spherical shape in solution. For a more accurate description of the observed diffusion behaviour, we therefore used the simplest deviation from spherical shape and modelled diffusing PAR chains as prolate ellipsoids with a short semiaxis ‘a’ and a long semiaxis ‘b’ (Figure 2E and F). For prolate ellipsoids, diffusion can be described by friction coefficients ${f}_\parallel$ and ${f}_ \bot$ for diffusion parallel or vertical to the long semiaxis. The overall translational diffusion coefficient D is then given by (67)


\begin{eqnarray*} {\rm{\ }}{D}_{ellipsoid} = \ {k}_BT\left[ {\frac{1}{{3{f}_\parallel }} + \frac{2}{{3{f}_ \bot }}} \right] \end{eqnarray*}

where ${f}_\parallel = 6\pi {\eta }_m\ r_\parallel ^{eff}$ and ${f}_ \bot = \ 6\pi {\eta }_mr_ \bot ^{eff}$. Here, $r_\parallel ^{eff}$ and $r_ \bot ^{eff}$ are hydrodynamic radii of spheres, which would exhibit the same diffusion behaviour. They are


\begin{eqnarray*} {\rm{\ }}r_\parallel ^{eff} = \ \frac{{8a}}{3}{\left[ { - \ \frac{{2H}}{{{H}^2 - 1}} + \frac{{2{H}^2 - 1}}{{{{\left( {{H}^2 - 1} \right)}}^{3/2}}}ln\left( {\frac{{H + \sqrt {{H}^2 - 1} }}{{H - \sqrt {{H}^2 - 1} }}} \right)} \right]}^{ - 1} \end{eqnarray*}

\begin{eqnarray*} {\rm{\ }}r_ \bot ^{eff} = \ \frac{{8a}}{3}{\left[ {\frac{H}{{{H}^2 - 1}} + \frac{{2{H}^2 - 3}}{{{{\left( {{H}^2 - 1} \right)}}^{3/2}}}ln\left( {H + \sqrt {{H}^2 - 1} } \right)} \right]}^{ - 1} \end{eqnarray*}

where $H\ = \ b/a$ is the aspect ratio of the ellipsoid (i.e. for a sphere the aspect ratio is 1) (68). The expected molecular masses of the respective PAR chains were calculated according to chain length assessments from the sequencing gels (Figure 2A and Table 1). To evaluate the aspect ratios of the diffusing PAR chains, we measured the diffusion times of the fluorescently labelled PAR chains using FCS and compared the results to those determined by a calibration curve, relating diffusion times to known molecular masses of the used standard proteins (Supplementary Figure S2). Since the calibration curve was obtained using largely globular proteins, this comparison yielded a diffusion time τ_calc_ assuming a spherical shape also for the PAR chain. This allowed us to grossly take the solvent shell into account. The observed difference between the experimentally determined diffusion time τ_exp_ and the calculated one τ_calc_ was then attributed to the deviation of the solvated PAR chain from spherical shape. As outlined above, an ellipsoid was used as a better approximation to the PAR chain shape. Its aspect ratio was obtained by adjusting its value in such a way that the corresponding friction coefficients ${f}_\parallel$ and ${f}_ \bot$ lead to a coincidence of τ_exp_ and τ_calc_ (Table 1). In this process, the volume of the ellipsoids is maintained. In general, the expression *T*(*H*) that describes the deviation of diffusion times between spherical and prolate ellipsoidal objects is given by


$$\begin{eqnarray*}T\ \left( H \right) & = & \ \ \frac{{{\tau }_{ellipsoid}}}{{{\tau }_{sphere}}} = \ \ \frac{{{D}_{sphere}}}{{{D}_{ellipsoid}}}\\ && = {\left[ {a \cdot \sqrt[3]{H} \cdot \left( {\frac{1}{{3*r_\parallel ^{eff}}} + \frac{2}{{3*r_ \bot ^{eff}}}} \right)} \right]}^{ - 1}\ \end{eqnarray*}$$

**Table 1.** Comparison of calculated (τ_calc._) and experimentally determined (τ_exp._) diffusion times. For definition of the aspect ratio *H*, please refer to text. HB indicates hyperbranched, WT, wildtype

			Diffusion time τ_exp_ [μs]		Aspect ratio H	
Number of ADP-ribose units per PAR chain	Calculated mass [kDa]	Diffusion time τ_calc._ [μs]	PAR^WT^	PAR^HB^	PAR^WT^	PAR^HB^
13	7.1	180	227	-	5.1	-
14	7.6	185	-	247	-	6.3
20	10.8	208	300	276	8.1	6.2
30	16.5	238	355	292	9	4.7
40	21.7	262	405	312	10	4.1
50	27.1	282	431	335	9.7	4.1

Figure 2E shows the correlation of the *T*(*H*) values with the respective aspect ratios *H* of the individual PAR fractions. The data demonstrate that the aspect ratios determined for PAR^WT^ chains depend only moderately on the chain length. The situation is different for hyperbranched PAR^HB^ chains. While the shortest PAR^HB^ chain investigated appears to be rather stretched, the longer the hyperbranched PAR^HB^ chains become, the more spherical they appear. This can be rationalized by the growing influence of the branching of the PAR chains in PAR^HB^ with increasing chain length on the 3D structure of the molecules. While it can be estimated that in the fraction of ‘PAR^WT, 50-mer^’ only one branching point was present per molecule, instead in ‘PAR^HB, 50-mer^’ molecules ∼6–7 branching points per chain were expected, probably resulting in a more spherical structure. This observation is important for understanding the binding of PAR to proteins as it reveals different spatial constraints for protein binding by PAR^WT^ and PAR^HB^.

### Interaction of p53 with its DNA response element RE_p21_

As mentioned above, in FCS measurements, the size of diffusing molecules is directly related to the diffusion times ${\tau }_D$. Since the binding between molecules results in changes of the observed hydrodynamic radius r, monitoring the diffusion times gives insight into molecular interactions (Figure 1). In many cases, fluorescence cross correlation spectroscopy (FCCS) has been used to investigate molecular interaction (69,70). This, however, requires the fluorescent labelling of both interacting species. Here, we employed standard FCS by single fluorescence labelling of the nucleic acid molecules only (i.e. DNA oligonucleotides and PAR), because we observed that fluorescence labelling of the main model protein used in this study (i.e. p53) led to quenching of the fluorophore and significantly affected its ability to tetramerize and to bind to its DNA consensus sequence.

**Figure 3.**
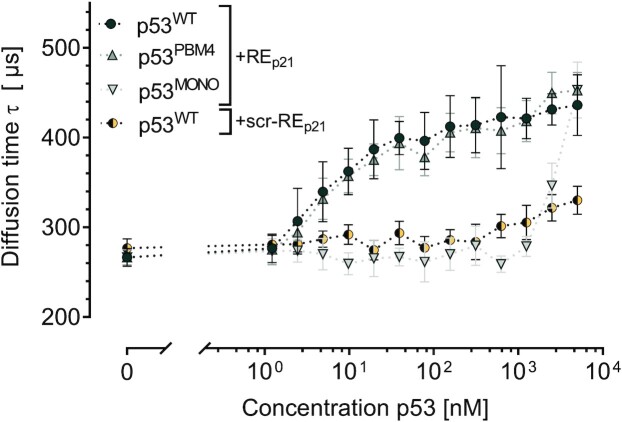
FCS-derived binding characteristics of different p53 variants to RE_p21E_ and RE_p21_^scr^. p53 concentration dependent diffusion times τ derived from FCS are displayed for p53^WT^, p53^PBM4^, and p53^MONO^ all measured at an oligonucleotide concentration of 10 nM. Values represent means ± SD of two independent experiments measured in technical triplicates (*n* = 6). The dotted lines between the data points only serve as guides to the eye.

To validate our experimental system, we analysed the previously well-studied interaction of p53 with its DNA response element RE_p21_, i.e. the 5′ consensus sequence site for binding of p53 via its DNA binding domain (DBD) within the promoter region of the p21 gene (71,72). Besides wildtype p53 (p53^WT^), we also studied the previously described p53^PBM4^ mutant, which carries four mutations in its C-terminal domain and which is deficient in PAR binding (21,22), as well as the tetramerization deficient mutant p53^MONO^ (L344P) (57). As sequence-specific DNA binding of p53 is mainly mediated via its DBD and only partially dependent on its C-terminal domain (CTD) (73,74), we expected strong binding of p53^WT^ and p53^PBM4^ to RE_p21_. As it is evident from Figure 3, p53^WT^ and p53^PBM4^ indeed bound RE_p21_ with high affinity. The *K*_d_-value for p53^WT^ obtained by curve fitting is 32 nM, which matches earlier published values (72,73,75).

To further test the specificity of the system with regards to p53–DNA interactions, we analysed the interaction of p53 with a version of RE_p21_, in which its nucleobase sequence has been completely scrambled, i.e. scr-RE_p21_. Here, only sequence-independent and much weaker DNA binding of p53^WT^ mainly mediated via its C-terminal domain was expected (21,22,76). This was confirmed by results of FCS measurements shown in Figure 3, which revealed the onset of binding at a p53^WT^ concentration of approximately 1 μM, i.e. at concentrations roughly 100 times higher than for wildtype RE_p21_. The tetramerization domain of p53 has a strong impact on the binding affinity of p53 to its DNA response elements (77) and potentially also to PAR (78). We therefore also analysed the binding of the monomeric mutant p53^MONO^, harbouring an L334P amino acid exchange in its tetramerization domain (57), to the RE_p21_. We found an onset of binding at high p53^MONO^ concentrations of approximately 1 μM (Figure 3), which confirms that the p53^MONO^ (L344P) variant is not able to bind to its consensus sequence efficiently as shown previously (57). In summary, our results are consistent with earlier findings concerning binding characteristics of p53 to its RE_p21_ DNA consensus sequence and thus demonstrate the suitability of our FCS approach for the investigation of interactions of p53 and other model proteins with structurally defined PAR chains.

### Impact of PAR chain length and branching on the interaction with p53

**Figure 4.**
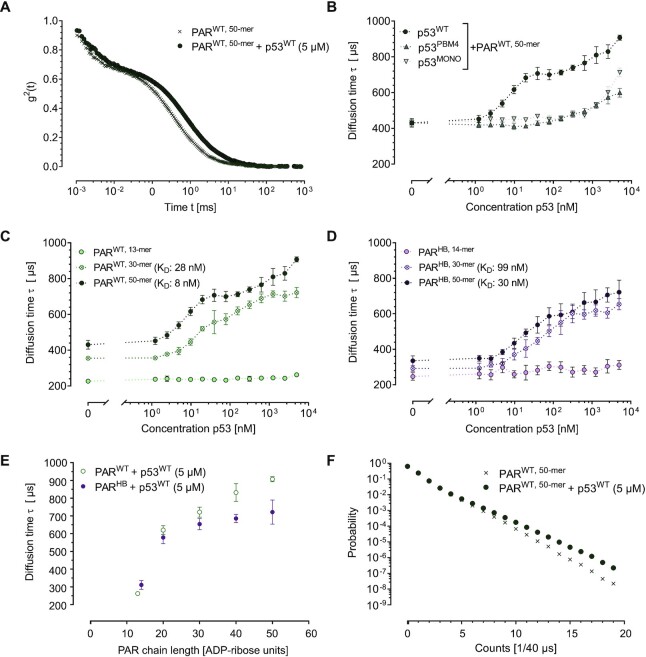
FCS-derived structure-specific PAR binding characteristics of different p53 mutants. (**A**) Second order autocorrelation functions of FCS measurements obtained for PAR50^WT^ alone or in presence of 5 μM p53^WT^. Diffusion times $\tau$ were obtained from fits to the data. Increasing diffusion times reflect increasing sizes of the diffusing species. (**B**) Diffusion times $\tau$ of 10 nM PAR^WT, 50-mer^ in Tris-buffer measured in presence of different p53 mutants as indicated. (**C**) Diffusion times $\tau$ of 10 nM PAR^WT^ of different chain length as indicated measured with increasing concentrations of p53^WT^. Data for the interaction of PAR^WT, 20-mer^ and PAR^WT, 40-mer^ are collected in Supplementary Figure S6a). (**D**) Diffusion times $\tau$ of 10 nM PAR^HB^ of different chain length as indicated measured with increasing concentrations of p53^WT^. Data for the interaction of PAR^HB, 20-mer^ and PAR^HB, 40-mer^ are collected in Supplementary Figure S6b). (**E**) Direct comparison of diffusion times $\tau$ of PAR^WT^ and PAR^HB^ of different chain lengths at a p53^WT^ concentration of 5 μM. Values represent means ± SD of two independent experiments measured in technical triplicates (*n* = 6). (**F**) Analysis of cluster formation between p53 and multiple PAR^WT, 50-mer^ molecules. Photon counting histograms from measurements of 10 nM PAR^WT, 50-mer^ alone or together with 5 μM p53^WT^ as indicated. Integration times are 40 μs, 20% of the PAR^WT, 50-mer^ chains were labelled with a single fluorophore. The dotted lines between the data points in (B), (C) and (D) only serve as guides to the eye.

After validating the experimental setup using p53 and RE_p21_, we studied the interaction characteristics of p53 with PAR of defined chain length and branching. We recorded the diffusion times of the fluorescently labelled PAR chains as a function of the concentration of unlabelled p53. The autocorrelation functions for PAR^WT, 50-mer^ alone or in presence of 5 μM p53^WT^ are shown in Figure 4A. The derived FCS binding curves show a clear shift to longer diffusion times due to the concentration dependent binding of p53^WT^ to PAR^WT, 50-mer^ (Figure 4B). Interestingly, after reaching a first plateau level at 50 nM p53^WT^, diffusion times again increased with higher p53^WT^ concentrations and did not reach a further plateau up to the highest tested p53 concentration of 5 μM. At the highest p53^WT^ concentrations tested, we recorded a diffusion time of 907 μs for the complexes formed between p53^WT^ and PAR^WT, 50-mer^. Earlier work showed that at these concentrations, p53^WT^ is expected to be present predominantly in its tetrameric form (22,73,75). A comparison with the size calibration data (Supplementary Figure S2) allowed us to estimate that at these concentrations, on average at least four p53 tetramers bound to one PAR^WT, 50-mer^. The question whether these large complexes might also have comprised several PAR chains, potentially forming a molecular network, is addressed below. For the first binding event, which was completed at approximately 100 nM p53^WT^, we calculated a *K*_d_-value of 8 nM by fitting the data. The finding that the *K*_d_ value for p53^WT^–PAR binding is similar or even lower than that of the p53^W^–RE_21_ interaction is in accordance with previous results showing that PAR and DNA act as competitive binding partner of p53, with PAR being able to efficiently abrogate p53–DNA binding (21,22,78).

As a comparison to the binding characteristics of p53^WT^, we also investigated the binding behaviour of the two p53 mutants p53^MONO^ and p53^PBM4^. In both cases, the binding to PAR was shifted to higher p53 concentrations and first formation of complexes was observed at concentrations of ∼500 nM p53 (Figure 4B). While in this case no plateau phases were reached even at the highest p53 concentrations tested and consequently no *K*_d_ values could be calculated, the observed binding behaviour suggests that the *K*_d_-values for p53^MONO^ and p53^PBM4^ are at least 100-fold higher than for p53^WT^. These results confirm that PAR-binding of p53 is mainly mediated via its CTD and can be strongly diminished by introducing distinct mutations (21,22). Moreover, our data suggest that p53 tetramerization is not only important for sequence-specific DNA binding, but also for efficient PAR binding. This is consistent with previous results showing that covalent PARylation of p53^MONO^ is strongly reduced compared to PARylation of p53^WT^, presumably due to reduced p53–PAR interaction, which serves a prerequisite for covalent PARylation of p53 by PARP1 (22).

We then analysed the influence of PAR chain length and branching on the interaction with p53^WT^. Of note, for both PAR^WT^ and PAR^HB^, a minimum PAR chain length of 20 monomeric units was necessary for binding of p53, since neither linear nor branched PAR^13/14-mer^ bound p53^WT^ efficiently in the tested concentration range. Previously, Fahrer et al. observed interaction of p53^WT^ with PAR^WT^ at a chain length of a 14-mer in surface plasmon resonance experiments and of a 16-mer in EMSA experiments, presumably caused by the influence of the specific biochemical conditions used in the individual experimental approaches on the binding characteristics. In the present study, p53-PAR interaction was first observed for PAR chains consisting of 20 monomeric units, while efficient binding was only detected for PAR chains with 30 and more monomeric units (Figure 4C–E). For both PAR types, i.e. PAR^WT^ and PAR^HB^, we observed increases in binding affinities with increasing chain length, which is in accordance with previous data for PAR^WT^ (18). Two important differences were observed in the interaction behaviour between PAR^WT^ and PAR^HB^ with p53^WT^. Firstly, for the same number of monomeric units in the PAR chains, we report larger diffusion times for PAR^WT^ than for PAR^HB^ at the final p53^WT^ concentration of 5 μM, the difference becoming more pronounced with increasing PAR chain lengths (Figure 4E). Since even the longest PAR chains are much smaller than p53 tetramers (PAR^50-mer^: ∼28 kDa versus p53 tetramer: ∼176 kDa), it is safe to assume that for large complexes the diffusion behaviour was still dominated by p53. This in turn implies that at the highest concentrations tested, significantly more p53^WT^ tetramers bound to PAR^WT^ than to PAR^HB^. A comparison to the size calibration data shows that in the first case approximately 11 tetramers bound, in the second ∼3. Secondly, for all PAR chain lengths tested, the diffusion times started to raise already at lower concentrations in the experiments with PAR^WT^ than in those using PAR^HB^. This indicates that the interaction between PAR^WT^ and p53^WT^ is stronger than the interaction between hyperbranched PAR^HB^ and p53^WT^. The calculated *K*_d_-values for the binding of p53^WT^ to the PAR chains are 28 nM (30-mer), 7 nM (40-mer), and 8 nM (50-mer) for PAR^WT^, and 99 nM (30-mer), 41 nM (40-mer) and 30 nM (50-mer) for PAR^HB^. For comparison, previously, Fahrer et al. reported *K*_d_ values of 250 nM for PAR^WT, 14–16mer^ and 130 nM for PAR^WT, 55–63mer^ as determined by biochemical EMSA as well as 3.4 nM for PAR^WT, 14–16mer^ by surface plasmon resonance studies (18). Fischbach et al. showed that addition of PAR resulted in an increased thermodynamic stability of p53 in a PAR-chain length dependent manner as measured by differential scanning fluorimetry (22). Furthermore, DNA-PAR competition experiments (i.e. via EMSA) revealed that longer PAR chains can compete more efficiently with the DNA-p53 binding than short PAR (22), again being in line with the higher affinity of p53 to long PAR chains as reported in the present study.

The observation that the binding of p53^WT^ to PAR^WT, 50-mer^ did not saturate for p53 concentrations ranging from 1 nM to 5 μM can either be explained by PAR chains binding large numbers of p53^WT^ tetramers or by the formation of a network in which several PAR chains were interconnected by binding to the same p53^WT^ tetramers at high p53 concentrations. To obtain further insight into this phenomenon, we acquired photon counting histograms (PCH) of 10 nM PAR^WT, 50-mer^ with and without 5 μM of p53^WT^ (Figure 4F, Supplementary Tables S2 and S3). PCH data show how often certain fluorescence emission intensities are recorded in a given experiment. Our biochemical PAR labelling strategy ensured that each labelled PAR chain only carries one fluorophore. Furthermore, the overall labelling degree for PAR^WT, 50mer^ was 20%. This means that each PAR chain can only contribute either 0 or the count rate of one individual dye molecule to the detected fluorescence. The detection of multiples of the average fluorescence emission intensity of an individual dye molecule would indicate the presence of several PAR^WT, 50mer^ chains in the detection volume. Since concentrations of PAR were chosen such that on average less than one freely diffusing PAR chain was present in the detection volume at a given time, this would show the formation of p53–PAR-complexes containing several PAR^WT, 50mer^ chains. In our PCH experiments, the sample additionally containing p53^WT^ exhibited a clear increase in the detection probability of higher count rates (Figure 4F) as compared to the pure PAR^WT, 50mer^ sample. The acquired data were fitted with a one and a two species model for the average brightness and the average number of particles in the detection volume. For the samples containing PAR^WT, 50-mer^ with p53^WT^, a fit model considering two species with different emission intensities had to be used to obtain acceptable fitting results (χ^2^(one species) = 15.1; χ^2^(two species) = 1.29). Considering the known labelling degree of the sample it was possible to estimate how many PAR chains were part of one PAR–protein complex. In addition, the size calibration curve (Supplementary Figure S2) allowed us to determine the mass of the complex. Together both pieces of information give insight into the total composition of the complexes. For the sample containing p53^WT^, we found a complex consisting of about 4 PAR^WT, 50-mer^ chains and about 5 p53^WT^ tetramers. This supports the hypothesis, that at high concentrations of p53^WT^, one p53^WT^ tetramer can bind to several PAR chains leading to the formation of a network of both components. This possible scenario of PAR-induced network formation of p53 is of particular interest in the context of findings showing that under certain conditions p53 can form liquid-phase condensates and amyloid-like fibrils (79–83). Furthermore, PAR was shown to trigger liquid-liquid phase separation processes of multiple intrinsically disordered and RNA-binding proteins at sites of DNA damage (84,85), as well as to be involved in pathological fibril biochemistry of α-synuclein (86). Interestingly, also the C-terminus of p53, which harbours the primary PAR binding site, represents an intrinsically disordered region (76). Associated with our results of a potential p53–PAR network formation and a potential role in liquid-liquid demixing processes, may be previous findings demonstrating that PAR binding can delay the temperature-induced irreversible formation of insoluble p53 aggregates (21). In future studies, it will be exciting to investigate a potential dynamic role of PARylation in p53 liquid condensate and amyloid-like fibril formation in more detail. Further, it will be important to clarify, if such a potential role may work in a similar manner as reported in a recent study by Rhine *et al.* Here, the authors demonstrated that very low concentrations of PAR (1 nM) are sufficient to trigger the liquid-liquid phase separation of the FUS protein at near physiological concentrations of 1 μM via transient interactions that trigger FUS oligomerization and condensation (87).

### Influence of PAR chain length and branching on the interaction with histone H1 and APLF

To understand how other established PAR binders interact with PAR of defined chain length and branching, we studied the interaction of PAR chains with histone H1 and the histone chaperone APLF.

**Figure 5.**
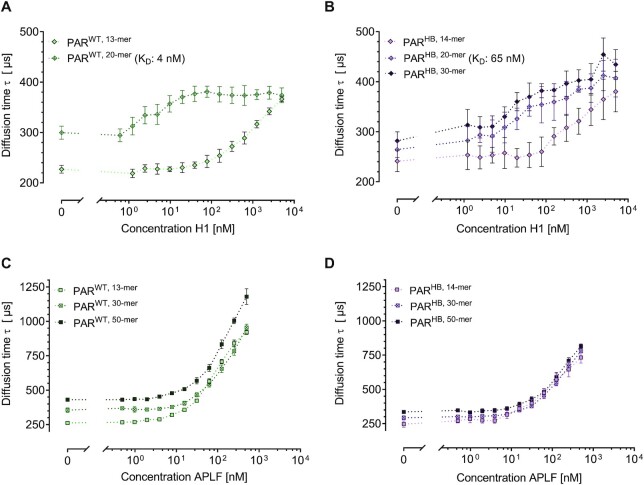
FCS-derived structure-specific PAR binding characteristics of histone H1 and APLF. (**A**) Diffusion times $\tau$ of PAR^WT^ of different chain length as indicated measured with increasing concentrations of H1. Mixing of H1 with longer PAR chains led to the formation of aggregates which precluded further investigations. (**B**) Diffusion times $\tau$ of PAR^HB^ of different chain length as indicated measured with increasing concentrations of H1. (**C**) Diffusion times $\tau$ of PAR^WT^ of different chain length as indicated measured with increasing concentrations of APLF. (**D**) Diffusion times $\tau$ of PAR^HB^ of different chain length as indicated measured with increasing concentrations of APLF. All measurements were performed at 10 nM PAR in Tris-buffer. Values represent means ± SD of two independent experiments measured in technical triplicates (*n* = 6). The dotted lines between the data points only serve as guides to the eye.

Figures 5A-B depict the results obtained for binding of H1 to PAR^WT^ and PAR^HB^ chains of different lengths. In contrast to p53, we found that H1 binds to PAR^WT, 13-mer^ and PAR^HB, 14-mer^, which are the shortest PAR chains tested. The data also revealed that H1 interaction with PAR^WT^ is chain-length dependent. Binding of H1 to PAR^WT, 20-mer^ is very strong with a *K*_d_ value of 5 nM, which is even below the dissociation constants determined for binding of p53 to the longest PAR chains tested, i.e. PAR^WT, 50-mer^. To the best of our knowledge this is the first report of a quantitative *K*_d_ value for histone H1-PAR interaction. A comparison between branched and linear PAR shows that PAR^HB, 20-mer^ and linear PAR^WT, 20-mer^ bound H1 with a similar strength (*K*_d_: 8 nM for PAR^HB, 20-mer^ versus 4 nM for PAR^WT, 20-mer^). Importantly, for PAR^WT, 20-mer^ a saturation of H1 binding was observed at a level of 1–2 bound H1 molecules per one PAR chain. Taken together with the finding that for both PAR^WT^ as well as PAR^HB^ a strong chain length dependency of H1 binding was observed, these data may suggest that several binding sites within a H1 molecule contribute to the H1–PAR interaction, thereby increasing the binding strength with increasing PAR chain length. The different stoichiometric PAR binding characteristics of p53 and histone H1 may be explained by the tetrameric vs. monomeric structure of p53 and H1, respectively, which allows p53 to form a network-like structure with PAR, which is not possible for histone H1. Interestingly, recording of binding curves for PAR chains longer than 20 subunits for PAR^WT^ and 30 subunits for PAR^HB^ was precluded because of aggregate formation of H1 in the presence of longer PAR chains. Based on our FCS data it is not possible for us to speculate about the nature of these aggregates. A common feature found for binding of p53 as well as of H1 was that PAR^WT^ binds the respective proteins stronger than PAR^HB^ of the same chain length. This is consistent with the notion of a potentially similar nature of the PAR binding modes for p53 and H1 via basic PBMs located in the intrinsically disordered C-terminal domains of the respective proteins.

Finally, we report the interaction of APLF and PAR with respect to potential PAR-structure-specific binding characteristics. In contrast to p53 and histone H1, which comprise basic PBMs, APLF harbours a tandem PBZ motif, consisting of two consecutive Cys_2_-His_2_ zinc fingers at position 376–404 (FI) and 418–440 (FII), which recognize independent ADP-ribose moieties within the polymer chain (49–51). Both zinc finger domains are connected via a linker region, which appears to be largely flexible providing some degree of freedom in the domain orientation (51). The PBZ represents a highly specialized PAR binding motif, which is present only in very few proteins, such as APLF, CHFR and SNM1A/DCLRE1A. The PBZ domains seem to specifically regulate the functions of these proteins in the DNA damage response and cell cycle progression (12). Interestingly, of the PBZ-containing proteins, only APLF contains a tandem PBZ motif. Eustermann et al. proposed that PAR interacts with a PBZ module via an adenine ring and a pyrophosphate group only when they occur on opposite sides of an intervening α(1→2) O-glycosidic bound, thereby enabling PAR-specific interactions (50). It has been reported that the tandem PBZ motif preferentially binds to branched PAR (25). This study by Chen et al. aimed to determine the binding site of APLF within wildtype PAR molecules by partial PARG digestion of APLF-bound PAR, subsequent sample work-up, and mass spectrometric determination of the di-ribosyladenosince (R_2_-Ado) versus ribosyladenosine (R-Ado) ratio. Upon PARG digestion of APLF-bound PAR, R_2_-Ado, which is indicative of PAR branching points, was highly enriched compared to R-Ado, which is indicative of the linear PAR structure (54).

In the present study, we directly compared binding characteristics of APLF with PAR of different chain lengths and branching frequencies in a comparative manner using the FCS set-up (Figure 5C, D). Consistent with previous reports (49,51,88), APLF bound PAR molecules with very high affinity (Figure 5C, D). Previous studies reported a *K*_d_ value of ∼1 nM for the APLF-PAR interaction as determined by SPR analyses using maximum protein concentrations up to 100 nM or up to 1 μM, respectively, with unfractionated, immobilized biotinylated PAR (49,51). In measurements of the present study, a determination of *K*_d_ values was not possible, since no plateau of diffusion times $\tau$ was reached at the maximum protein concentration. The very strong binding of APLF to PAR was also consistent with previous data showing that PAR-binding was abolished by mutation of both putative zinc-finger motifs, but not by mutation of a single of such motifs (49). Furthermore, Li *et al.* reported that the tandem PBZ domain displayed over a 1000-fold higher binding affinity compared to PBZ1 or PBZ2 alone (51). Based on those results and the findings that the PAR-binding affinity of the isolated PBZ1 peptide (*K*_d_: 520 nM) was almost 20-fold greater than that of PBZ2 (*K*_d_: 8.3 μM), Li *et al.* suggested that the first APLF ZF may serve as the primary high affinity ‘anchoring’ sites for PAR, and that the close proximity between the PBZ1 and PBZ2 leads to synergistic binding of PAR (51). Strikingly, in contrast to p53 and histone H1, we observed no chain length dependence of APLF–PAR interactions (Figure 5C-D), highlighting the different binding modes of the APLF PBZ domains as compared to the PBM-mediated PAR binding of p53 and histone H1. For short PAR chains, i.e. PAR^13/14-mer^, APLF was the strongest PAR binder of the three proteins tested, with an onset of binding at protein concentration of 10 nM. In comparison, no binding to p53 was observed for PAR^13/14-mer^ and the onset of binding for histone H1 and PAR^13/14-mer^ was observed at a protein concentration of 1 μM. For long PAR chains, i.e. PAR^50-mer^, the onset of PAR binding to p53 starts at lower protein concentrations (∼2 nM for p53 versus 10 nM for APLF), however, at higher protein concentrations significantly more APLF molecules bound to one PAR chain as was the case for p53. Indeed, our data suggest that numerous APLF molecules can bind to one PAR chain, which is evident by the finding that we did not observe a saturation of the binding curve at the highest APLF concentration tested (i.e. 500 nM), which excluded the determination of *K*_d_ values from our measurements. Similar to what has been observed for p53, these data hint at the formation of multivalent PAR–protein structures. On the molecular level, this implies either that each of the two PBZ motifs in one APLF molecule can bind separate PAR chains or that APLF molecules bound to a PAR chain can multimerize, thereby leading to multimeric complex or even network formation. In contrast to the study by Chen et al. (25), a preference for APLF binding to PAR^HB^ over PAR^WT^ is not evident from our analysis. Instead, in our setting, APLF bound linear PAR^WT^ with higher affinity than PAR^HB^. The reason for this discrepancy with the results reported by Chen *et al.* is not clear at present, however, while the study by Chen *et al.* provided indirect evidence for preferential binding of APLF to branched PAR, our study lacks direct evidence for a PAR-branching specific binding of APLF. Furthermore, different outcomes may be caused by differences in the experimental setups of both studies.

## CONCLUSION

Hundreds of proteins interact with PAR via an ever-growing list of PAR interaction motifs, thereby regulating their physico-chemical properties, sub-celluar localizations, enzymatic activities, or protein stability (10,12,14,16). Here, we chose a targeted, hypothesis-driven approach to characterize potential structure-specific interactions of PAR molecules of defined chain length and branching with three prime examples of PAR binding proteins, i.e. p53, histone H1 and APLF via FCS (Supplementary Figure S7). Our study reveals complex and structure-specific PAR–protein interaction characteristics (Table 2). For example, we quantitatively determined chain-length-specific binding characteristics for p53 and histone H1, as observed before with different methods (18,21,36), but chain length independent binding for APLF (Table 2). Concerning the biochemical basis of the chain length specificities, it can be speculated that multivalent interactions of long chains of PAR within the interacting protein may both increase affinity and specificity via consecutive binding to several binding motifs (12). At least for p53, three other binding sites were reported in peptide-based studies (78) in addition to its main binding site located in the CTD (22). While our previous analysis showed that all of those additional three potential binding sites are not able to mediate PAR binding on their own (22), it still may be possible that they participate in chain length dependent PAR binding as secondary binding modules after the PAR molecule has been anchored at p53 via its primary PBM located within the CTD. In general, such chain length dependent binding strongly supports the existence of distinct biological ‘information’ hidden in the heterogeneous chain lengths of PAR molecules (10). For example, the finding that p53 and histone H1 did not or only weakly bind to PAR of very short chain length, is particularly interesting when considering recent cellular findings of cells expressing a PARP1 mutant that synthesizes very short polymer (i.e. PARP^Y986S^). These cells were more sensitive to genotoxic camptothecin treatment than cells expressing wild-type PARP1 and displayed strongly diminished recruitment of the base excision repair factor XRCC1 to sites of laser-induced DNA damage (17).

**Table 2.** Summary of PAR^WT^ binding characteristics of p53, histone H1, and APLF. N.D. indicates not determined. PAR^WT^ chain lengths are the ones for the smallest determined K_d_ values

Protein	*K*_D_ value [nM]	Onset of PAR binding (nM)	Chain length dependency?	Preference for PAR^WT^ or PAR^HB^?	Stoichiometry [PAR:protein]	Network formation?
p53	7 nM (PAR^WT, 40-mer^)	2 nM	Preference for longer PAR chains	Slight preference for PAR^WT^ over PAR^HB^	Binding of multiple protein molecules per PAR chains	Likely
H1	5 nM (PAR^WT, 20-mer^)	2 nM	Preference for longer PAR chains	Slight preference for PAR^WT^ over PAR^HB^	∼1:1–2	Unlikely
APLF	N.D.	10 nM (for all PAR chain lengths)	No chain length preference	Slight preference for PAR^WT^ over PAR^HB^	Presumably binding of multiple protein molecules per PAR chains possible.	Potentially

Apart from PAR chain length, recent studies by Chen *et al.* (25) and Aberle *et al.* (17) point to branching specific cellular roles of PAR and Chen *et al.* reported that APLF exhibits a preference for branched PAR. We therefore had hypothesized that APLF may represent a well-suited model protein to study branching specific PAR binding characteristics using FCS. This hypothesis, however, could not be validated in the present study, since for APLF, as well as p53 and histone H1, the interaction with linear PAR^WT^ was moderately stronger as compared to binding to hyperbranched PAR (Table 2). While this leaves the identification and validation of branching-specific PAR binders open for future studies, the FCS approach presented here provides a powerful tool to validate potential PAR-structure-specific interactions with PAR readers to be identified.

Finally, the finding of distinct concentration-dependent binding courses of the three PAR binders tested have interesting biological implications. While the interaction of PAR with histone H1 appears to happen largely on a bi-molecular basis, instead for p53, and potentially also APLF, our data are consistent with the formation of multi-valent protein-PAR network structures (Table 2). In a cellular context, such potential multi-molecular protein-PAR complexes may play essential roles in liquid-liquid demixing processes (16,85). In this regard, it has been shown that PAR can function as a seed for the formation of biomolecular condensates at sites of DNA damage (10,16,84,85). In general, there is now increasing evidence that PAR may act as general determinant to regulate the dynamics and compartmentalization of protein assemblies via multivalent, non-covalent interactions in response to genotoxic stress in a PAR-structure- and concentration-specific manner (10,16,84,85). Furthermore, results from the present and several other studies reviewed in (10,12) indicate that protein-specific and PAR-structure-specific binding modes have evolved to fit the purpose of the respective biochemical functions and biological contexts. In general, the FCS set-up as presented in this study, represents a well suitable approach for a detailed and quantitative understanding of PAR–protein interaction characteristics in solution-based setting at near physiological buffer conditions. This can be instrumental for follow-up studies to unravel the individual contributions of distinct PAR readers and binding modes to PARylation-mediated cellular and organismic processes.

## DATA AVAILABILITY

The data underlying this article are available in the article and in its online supplementary material.

## Supplementary Material

gkac1235_Supplemental_File
