# Supplementary material for: Influence of chain length and branching on poly(ADP-ribose)–protein interactions
Source: Nucleic Acids Res. 2023 Jan 10;51(2):536–52. doi: 10.1093/nar/gkac1235 (PMC9881148; doi:10.1093/nar/gkac1235)
Supplement: gkac1235_Supplemental_File [file gkac1235_supplemental_file.docx]

**Supporting information:** **Influence of chain length and branching on poly(ADP-ribose)-protein interactions**

Tobias Löffler^1^ ^§^, Annika Krüger^2^ ^§^, Peyman Zirak^1^, Martin J. Winterhalder^1^, Anna-Lena Müller^2^, Arthur Fischbach^2^, Aswin Mangerich^2, 3^ ^#^ * and Andreas Zumbusch^1 #^ *

^1^ Department of Chemistry, Universität Konstanz, Konstanz, D-78457, Germany

^2^ Department of Biology, Universität Konstanz, Konstanz, D-78457, Germany

^3^ Institute of Nutritional Science, University of Potsdam, D-14558 Nuthetal, Germany

^§^ Shared first authors

# Shared senior authors

* To whom correspondence should be addressed:

AM: Tel: +49 (0)33200 88 5301; Email: mangerich@uni-potsdam.de

AZ: Tel: +49 (0)7531-882027; Email: andreas.zumbusch@uni-konstanz.de

**1.) PAR characterization**


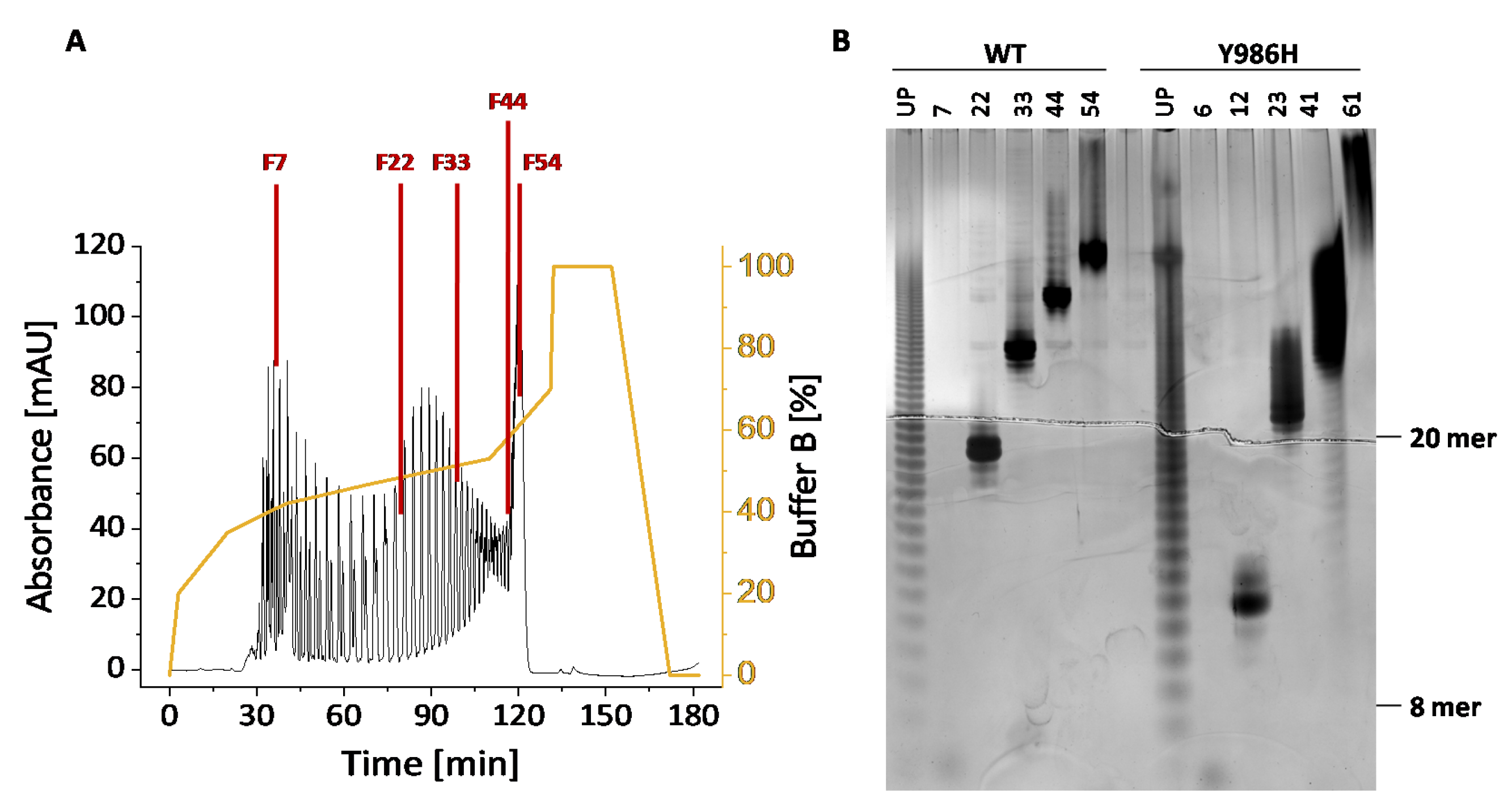

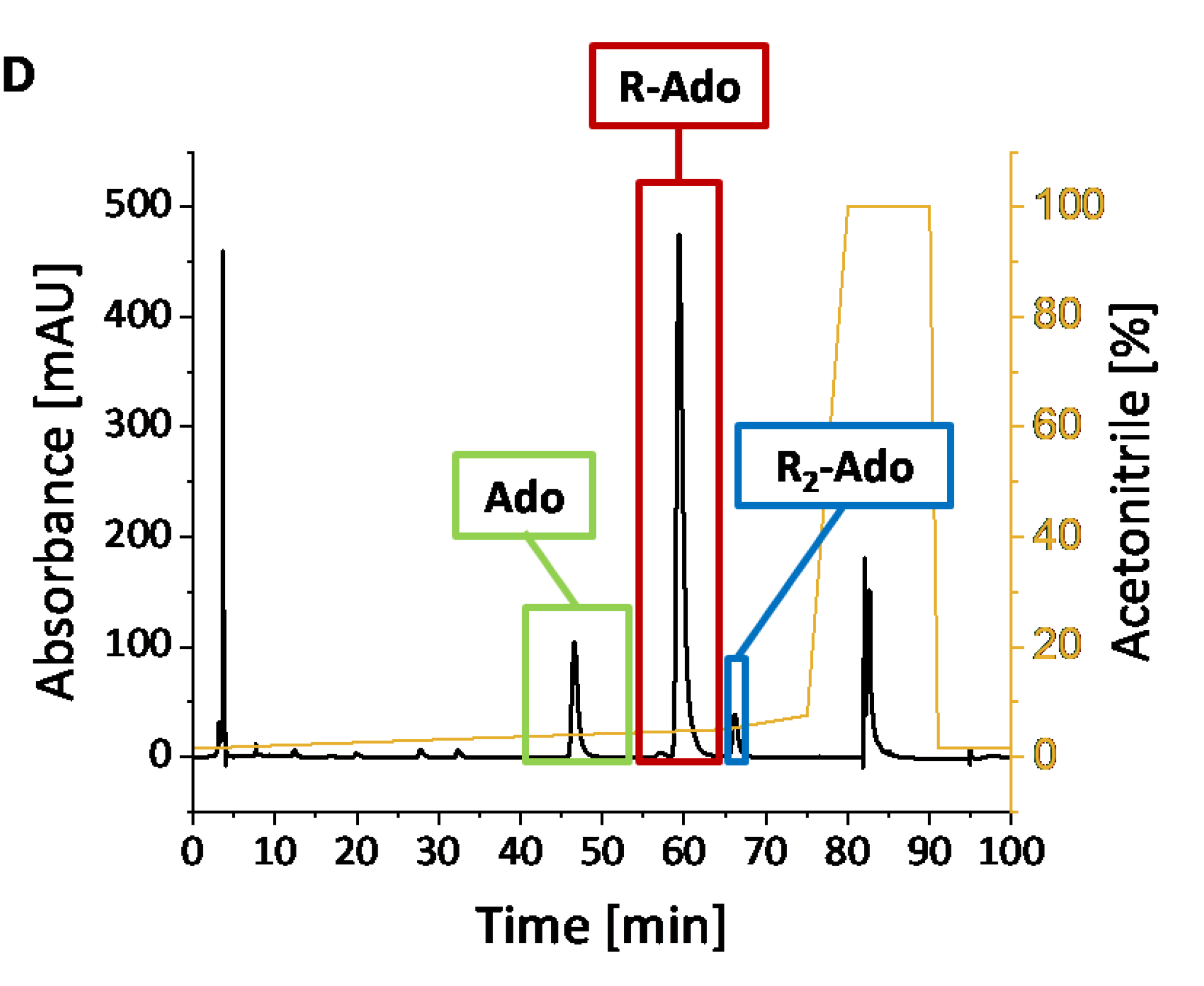


a)

b)

c)

e)

d)

f)

**Figure S1**. Supplementary data for the characterization of PAR molecules. a) Representative HPLC chromatogram of PARWT fractionation. Fractions of PAR analysed by sequencing gel and mass spectrometry are highlighted. b.) PAGE analysis of PARWT and PARHB fractions with subsequent silver staining. UP = unfractionated PAR. c) Representative HPLC chromatogram of digested PARHB showing specific peaks of Ado, R-Ado, and R2-Ado, which were purified to obtain respective standard compounds. d-f) Respective UPLC-MS/MS standard curves of R2-Ado, R-Ado, and Ado. AUC: area under the curve.

| **Step** | **Time [min]** | **H_2_O [%]** | **Acetonitrile [%]** |
| --- | --- | --- | --- |
|  |  |  |  |
| 1 | 0 | 98.5 | 1.5 |
| 2 | 65 | 95 | 5 |
| 3 | 75 | 92.5 | 7.5 |
| 4 | 80 | 0 | 100 |
| 5 | 90 | 0 | 100 |
| 6 | 91 | 98.5 | 1.5 |
| 7 | 100 | 98.5 | 1.5 |

**Table S1.** HPLC gradient for the separation of Ado, R-Ado, and R_2_-Ado were separated via HPLC.

**2.) Calibration of molecular masses derived from diffusion times**

In this work, we used diffusion times determined in FCS measurements to estimate the molecular masses of the diffusion molecules and to determine the aspect ratios of large molecular aggregates. To obtain a calibration curve, we measured diffusion times of fluorescently labelled globular proteins commonly used as size standards in size exclusion chromatography.

**
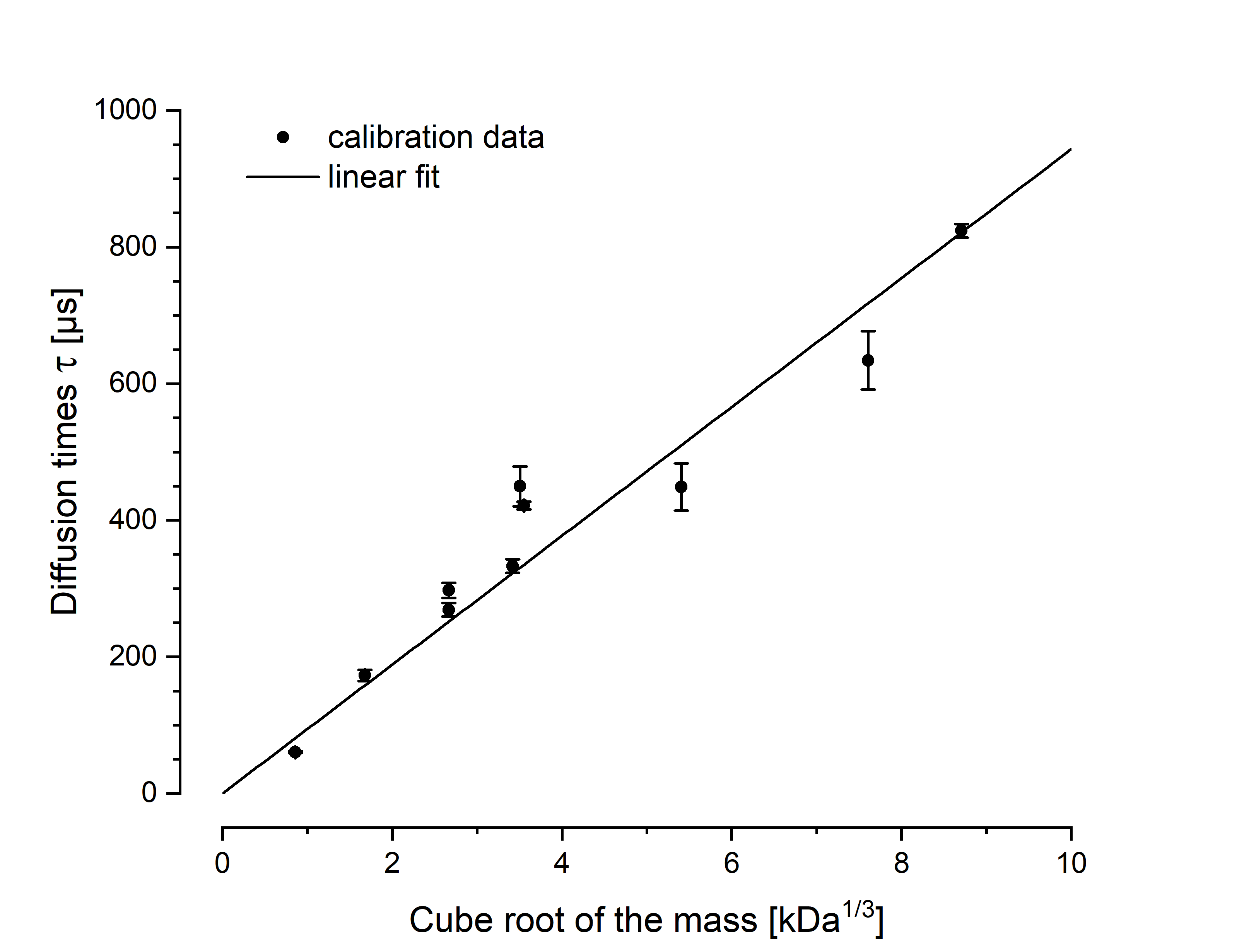
**

|  | mass [kDa] | tau [µs] | SEM [µs] | Cube root(m) |
| --- | --- | --- | --- | --- |
| Atto655 | 0.6 | 60.7 | 0.51 | 0.86 |
| Mp53tet | 4.7 | 173.0 | 2.73 | 1.68 |
| p21_scr_ | 19 | 297.5 | 1.86 | 2.67 |
| p21 | 19 | 269.0 | 1.67 | 2.67 |
| Aldolase monomer | 39.5 | 332.9 | 3.34 | 3.41 |
| Ovalbumin | 43 | 449.7 | 9.64 | 3.50 |
| p53M-wt | 44.7 | 421.7 | 1.92 | 3.55 |
| Aldolase | 158 | 448.7 | 11.51 | 5.41 |
| Ferritin | 440 | 634.2 | 14.33 | 7.61 |
| Thyroglobulin | 660 | 824.0 | 3.28 | 8.71 |

**Figure S2**. Calibration data for the calculation of molecular masses from diffusion times determined by FCS.

**3.) Determination of K_d_ values**

The determination of the dissociation constants K_d_ was based on FCS measurements of the dilution series (see Methods and Materials section).

a) The autocorrelation function for two diffusing species 1 and 2 (assuming the same brightness per particle) can be written as

$$g^{\left( 2 \right)}\left( \tau\right)=\frac{1}{\left\langle N \right\rangle}\left( f_{1}\cdot\tilde{g}_{1}^{\left( 2 \right)}\left( \tau\right)+f_{2}\cdot\tilde{g}_{2}^{\left( 2 \right)} \right)$$

where $\left\langle N \right\rangle=\left\langle N_{1} \right\rangle+\left\langle N_{2} \right\rangle$ is the overall number of particles, f_1_ and f_2_ are the fractions of the two species contributing to $g^{\left( 2 \right)}\left( \tau\right)$, and $\tilde{g}_{i}^{\left( 2 \right)}\left( \tau\right)$ are the autocorrelation functions of each species multiplied with its number of particles ( $\tilde{g}_{i}^{\left( 2 \right)}\left( \tau\right)=\left\langle N_{i} \right\rangle\cdot g_{i}^{\left( 2 \right)}\left( \tau\right)$ ).

b) The dissociation of a complex was considered as an equilibrium reaction of the species A and P

$$\left[ AP \right]\rightleftharpoons\left[ A \right]+\left[ P \right]$$

with the dissociation constants

$$K_{d}=\frac{\left[ A \right]\cdot\left[ P \right]}{\left[ AP \right]}\left( 1 \right)$$

The fraction f of complexes (e.g. bound molecules) is given by

$$f=\frac{\left[ AP \right]}{\left[ AP \right]+\left[ A \right]+\left[ P \right]}$$

Molecules A were considered to be the partially fluorescently labelled species. The concentration of A was kept constant at 10nM while the concentration [P] was varied. While the labelling degree differed, the fraction of detected bound molecules A* can be approximated by

$$f\approx\frac{\left[ A^{*}P \right]}{\left[ A^{*}P \right]+\left[ P \right]}\left( 2 \right)$$

as

$$\left[ P \right]\gg\left[ A^{*} \right]$$

c) The combination of a) and b) describes the measured FCS curves for the dilution series

$$g^{\left( 2 \right)}\left( \tau\right)=\frac{1}{\left\langle N \right\rangle}\left( \frac{1-f_{2}}{\left( 1+\frac{\tau}{\tau_{1}} \right)}+\frac{f_{2}}{\left( 1+\frac{\tau}{\tau_{2}} \right)} \right)$$

with fractions f_1_ and f_2_ of bound and unbound labelled molecules and $f_{1}=1-f_{2}$. Correlation functions were calculated using SymphoTime (PicoQuant).

To reduce the number of free parameters diffusion times τ_1_ and τ_2_ were determined as follows. Diffusion time τ_1_ for the unbound labelled molecule A was obtained by fitting the FCS curve with [P] = 0, whereas diffusion time τ_2_ was calculated from τ_1_ and the increase of diffusion time expected by complex formation. Like this the correlation functions $g^{\left( 2 \right)}\left( \tau\right)$ at different concentrations [P] could be fitted with $\left\langle N \right\rangle$ and f_2_ as free parameters using the ‘curve fitting tool’ from MATLAB (version R2018b, MathWorks) by using the ‘Trust-region’ algorithm from the ‘NonlinearLeastSquares’ method.

d) Combining equation (1) with equation (2) gives an expression to determine the dissociation constant K_d_.

$$\left[ AP \right]=\frac{f\cdot\left[ A \right]}{1-f}$$

$$\frac{K_{d}}{1-f}=\frac{\left[ A \right]\cdot\left[ P \right]}{f\cdot\left[ A \right]}$$

$$f=\frac{\left[ P \right]}{K_{d}+\left[ P \right]}$$

Fractions f_2_ of bound molecules A corresponding to the fractions f in the equilibrium equation (see above) are plotted versus the concentration [P]. Finally, the curves $f\left( \left[ P \right] \right)$were fitted to yield the dissociation constants K_d_ for the binding equilibrium (‘MATLAB, version R2081b, MathWorks).

**4.) Exemplary data for the determination of K_d_ values**


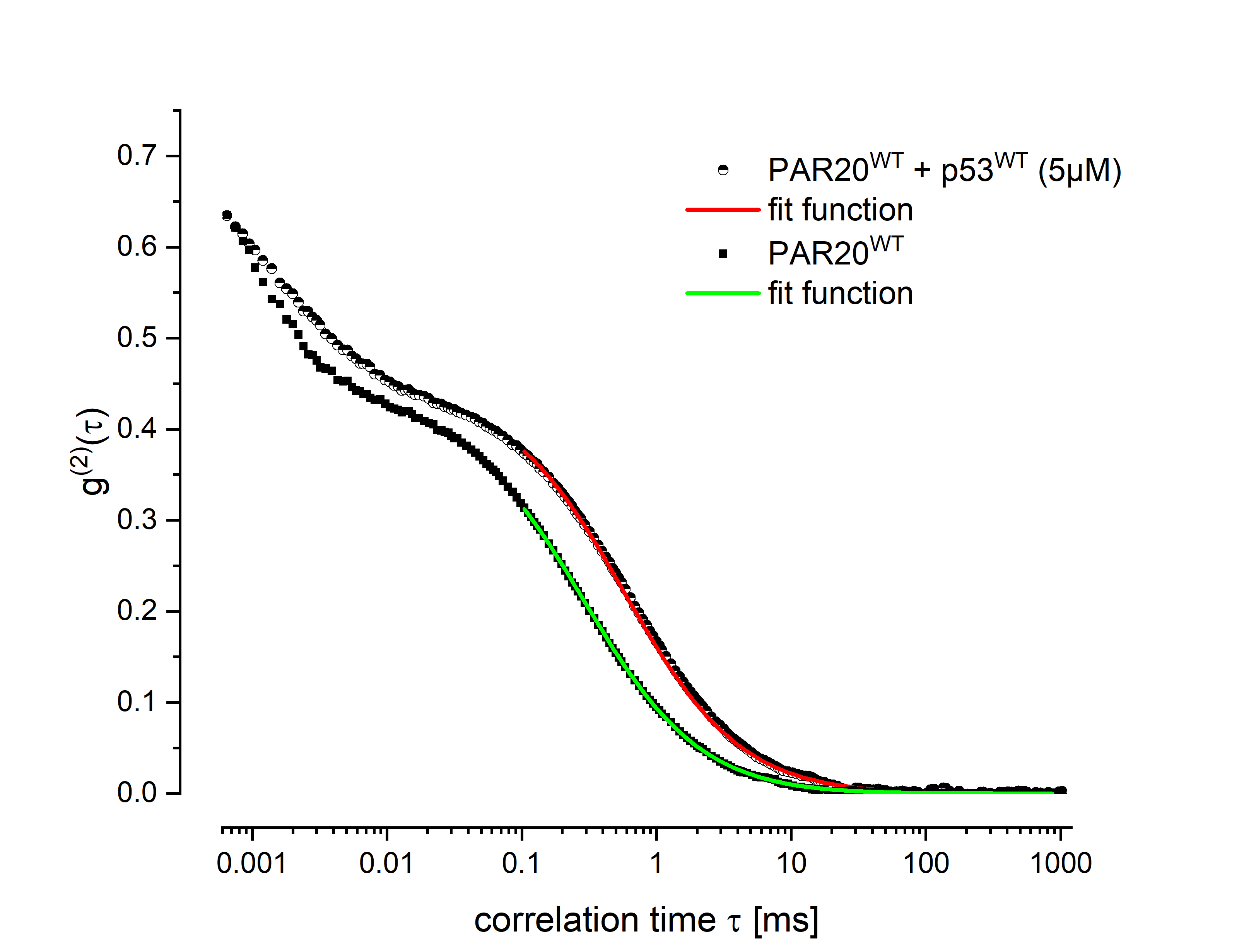


**Figure S3.** Exemplary FCS data and fitting curves. Shown are FCS data for the binding experiment of PAR20^WT^ with p53^WT^. Correlation functions and diffusion time τ_1_ of PAR20^WT^ were calculated using SymphoTime (PicoQuant). Resulting fitting curves from SymphoTime are shown.


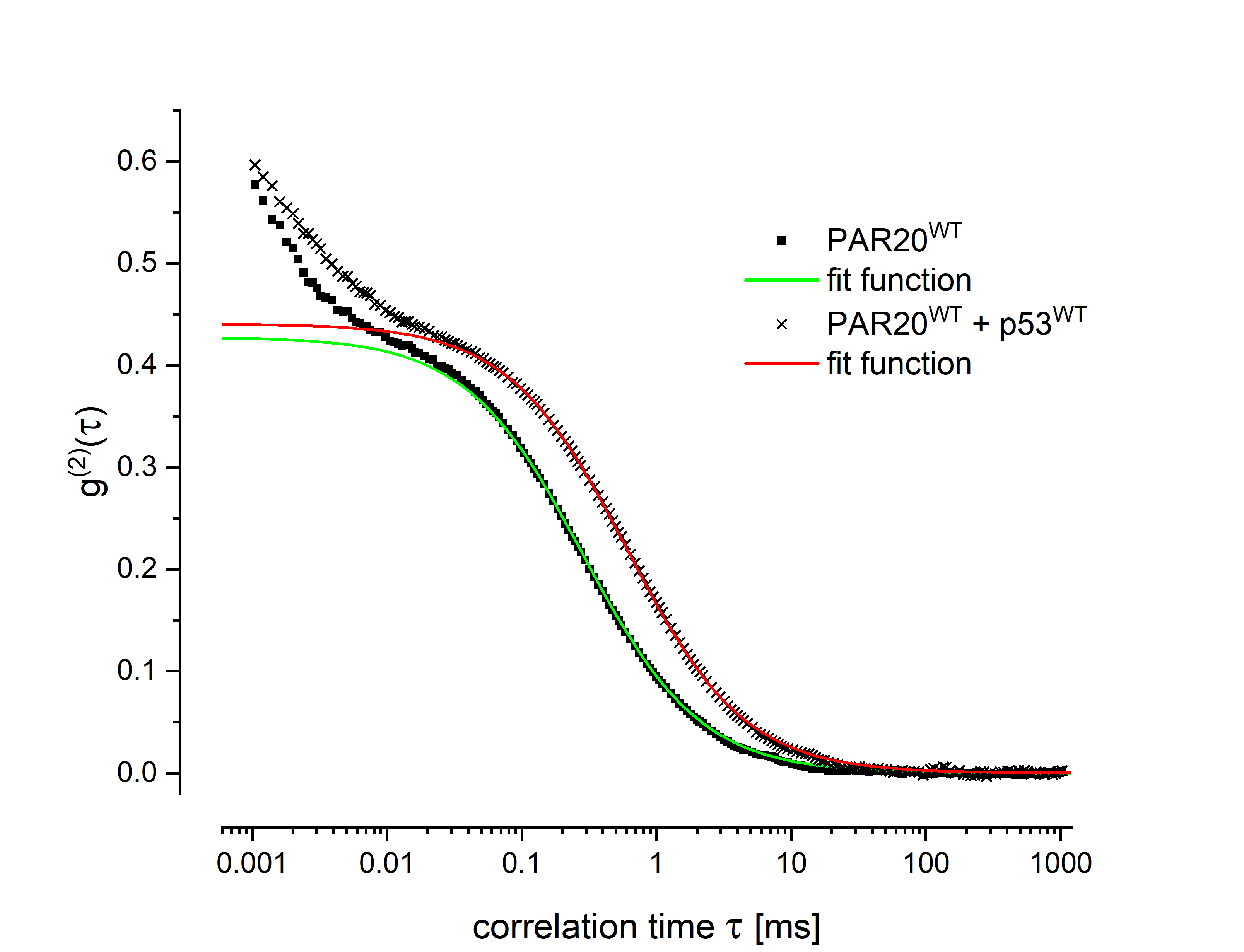


**Figure S4.** FCS data and fitting curves for binding experiment of PAR20^WT^ with p53^WT^. Diffusion times were used to fit the correlation curves resulting in fractions of bound PAR20^WT^. Fitting was done using MATLAB (MathWorks). Note that the short time behaviour is due to triplet formation of the fluorophores which was not considered in the fitting functions.


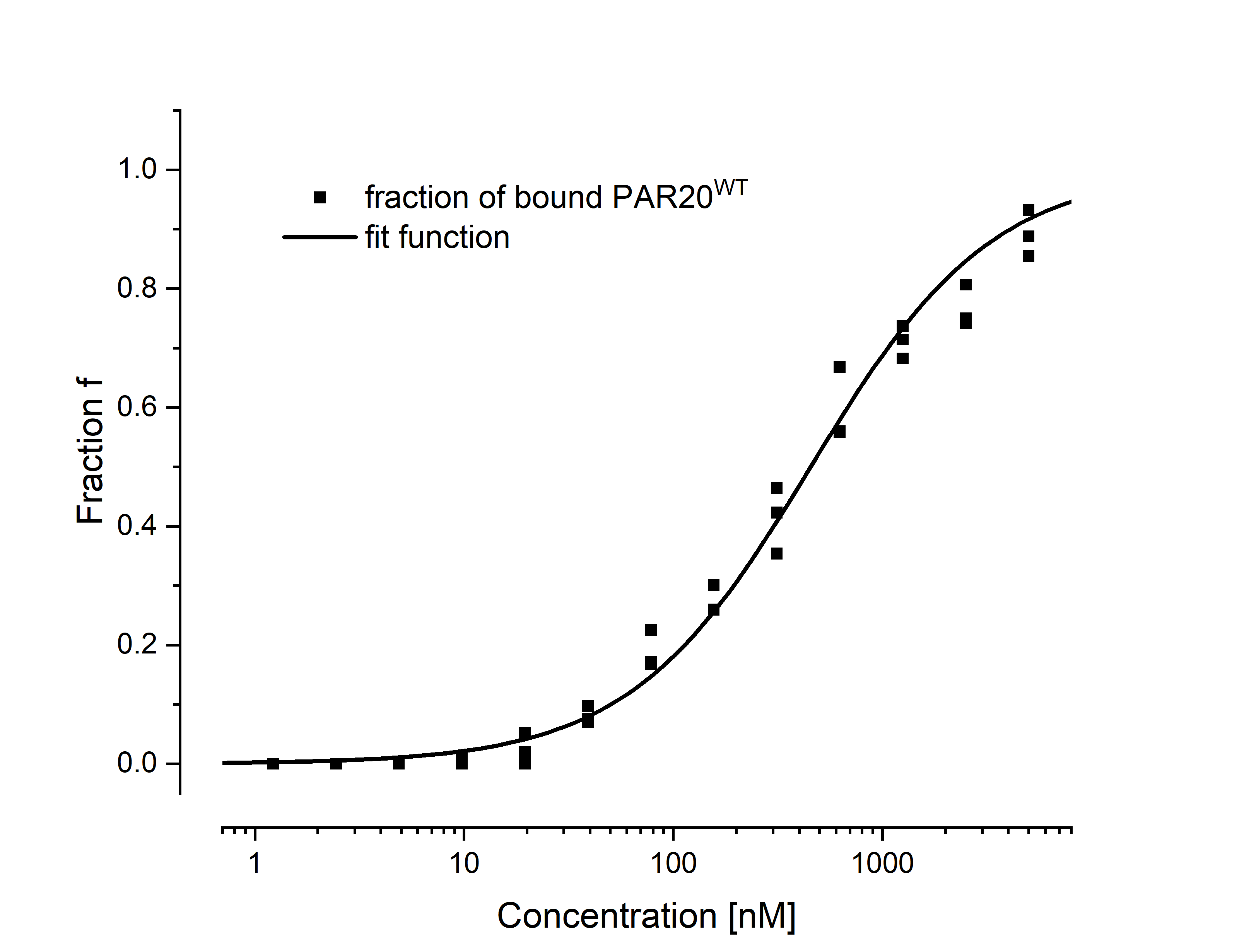


**Figure S5.** Fractions f of bound PAR20^WT^ as function of total PAR20^WT^ concentration are shown. The values were derived using the procedure outlined in 3.) The data shown are technical triplicates of one biological duplicate. The dissociation constant K_d_ was then determined by fitting (MATLAB, MathWorks). For further details see 3.)

**5.) FCS-derived structure-specific PAR binding with p53^WT^**

As mentioned in the main text, interactions between PAR and p53^WT^ were also measured for PAR 20-mers and 40-mers. In the main text, for clarity only data for 10-mers, 30-mers, and 50-mers were depicted. The figures below also show the data for PAR 20-mers and 40-mers.


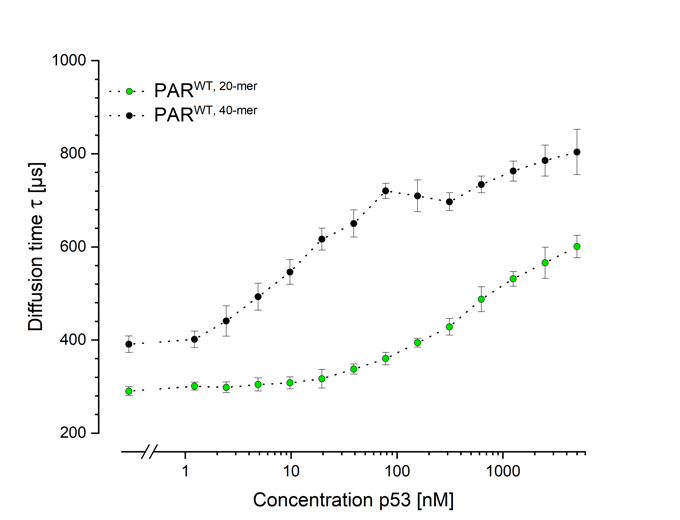

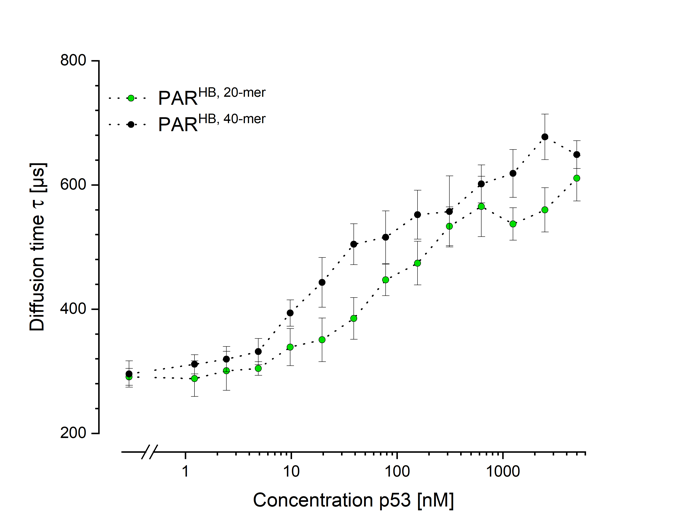


**b)**

**a)**

**)**

**Figure S6.** a) Diffusion times $\tau$ of 10 nM PAR^WT^ of different chain length as indicated measured with increasing concentrations of p53^WT^. b) Diffusion times $\tau$ of 10 nM PAR^HB^ of different chain length as indicated measured with increasing concentrations of p53^WT^.

**6.) Fit parameters for the calculation of the composition of large PAR50-wt and p53-wt complexes from photon counting histogram data**

The fitting procedures and the model used are described in detail in the main text. The lower two lines in **Table S2** show the quality of the fitting results for assuming only one species (upper line) or two species (lower line). Here, as explained in the main text, one species corresponds to complexes containing only one fluorophore whereas the second species groups all complexes carrying two or more fluorophores.

|  | N_1_ | +/- | q_1_ | +/- | N_m_ | +/- | q_m_ | +/- | χ^2^ | +/- |
| --- | --- | --- | --- | --- | --- | --- | --- | --- | --- | --- |
| PAR50-wt | 0.97 | 0.03 | 66137 | 245 | - | - | - | - | 4.42 | 0.33 |
| PAR50-wt + 5µM p53-wt | 0.85 | 0.03 | 86150 | 2410 | - | - | - | - | 15.11 | 2.50 |
| PAR50-wt + 5µM p53-wt | 0.67 | 0.03 | 49893 | 10161 | 0.28 | 0.03 | 109400 | 6151 | 1.29 | 0.23 |

**Table S2:** Parameters obtained for fitting the PCH data.

These data indicate the presence of species carrying multiple fluorophore labels (**Table S2**). Based on these data, we calculate the mean composition of the PAR50^WT^-p53^WT^ aggregates using the approach outlined in the main text.

|  | N_1_/N_m_ | q_m_/q_1_ | labelling degree | PAR units from N | PAR units from q | mass complex [kDa] | p53 tetramers |
| --- | --- | --- | --- | --- | --- | --- | --- |
| PAR50 + 5µM p53 | 2.42 | 2.19 | 0.20 | 3.80 | 4.00 | 940.00 | 4.91 |

**Table S3.** Composition of PAR50^WT^-p53^WT^ aggregates calculated using the fitting parameters shown in **Table S2**.

**7.) Comparison of binding data of the different PAR molecules and the proteins investigated**

**Figure S7** compiles data presented and discussed in the main text such that the different binding behavior of the various PAR molecules to the three proteins investigated becomes possible.


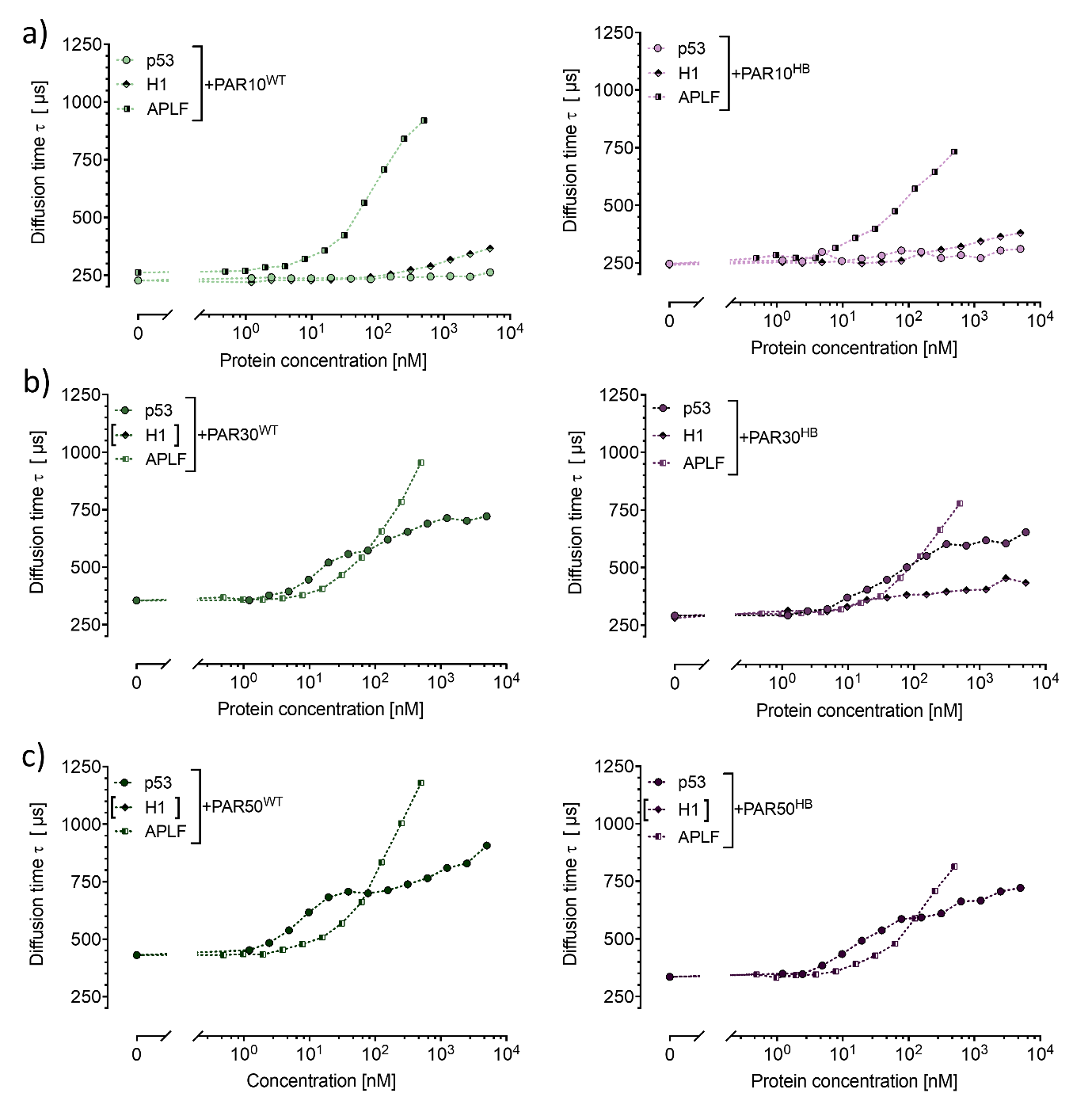


**Figure S7.** Binding behaviour of various PAR molecules to the three different proteins investigated.
